# A novel promising laccase from the psychrotolerant and halotolerant Antarctic marine *Halomonas* sp. M68 strain

**DOI:** 10.3389/fmicb.2023.1078382

**Published:** 2023-02-10

**Authors:** Melissa Bisaccia, Elisa Binda, Elena Rosini, Gabriella Caruso, Ombretta Dell'Acqua, Maurizio Azzaro, Pasqualina Laganà, Gabriella Tedeschi, Elisa M. Maffioli, Loredano Pollegioni, Flavia Marinelli

**Affiliations:** ^1^Department of Biotechnology and Life Sciences (DBSV), University of Insubria, Varese, Italy; ^2^Institute of Polar Sciences (CNR-ISP), National Research Council, Messina, Italy; ^3^Institute of Polar Sciences (CNR-ISP), National Research Council, Venice, Italy; ^4^Department of Biomedical and Dental Sciences and Morphofunctional Imaging (BIOMORF), University of Messina, Messina, Italy; ^5^Department of Veterinary Medicine and Animal Science (DIVAS), University of Milan, Milan, Italy; ^6^Cimaina, University of Milan, Milan, Italy

**Keywords:** Antarctica, laccase, *Halomonas* sp., marine biofilm, cold-adaptation, extremophile bacteria

## Abstract

Microbial communities inhabiting the Antarctic Ocean show psychrophilic and halophilic adaptations conferring interesting properties to the enzymes they produce, which could be exploited in biotechnology and bioremediation processes. Use of cold- and salt-tolerant enzymes allows to limit costs, reduce contaminations, and minimize pretreatment steps. Here, we report on the screening of 186 morphologically diverse microorganisms isolated from marine biofilms and water samples collected in Terra Nova Bay (Ross Sea, Antarctica) for the identification of new laccase activities. After primary screening, 13.4 and 10.8% of the isolates were identified for the ability to oxidize 2,2′-azino-bis (3-ethylbenzothiazoline-6-sulfonic acid) (ABTS) and the dye azure B, respectively. Amongst them, the marine *Halomonas* sp. strain M68 showed the highest activity. Production of its laccase-like activity increased six-fold when copper was added to culture medium. Enzymatic activity-guided separation coupled with mass spectrometry identified this intracellular laccase-like protein (named Ant laccase) as belonging to the copper resistance system multicopper oxidase family. Ant laccase oxidized ABTS and 2,6-dimethoxy phenol, working better at acidic pHs The enzyme showed a good thermostability, with optimal temperature in the 40–50°C range and maintaining more than 40% of its maximal activity even at 10°C. Furthermore, Ant laccase was salt- and organic solvent-tolerant, paving the way for its use in harsh conditions. To our knowledge, this is the first report concerning the characterization of a thermo- and halo-tolerant laccase isolated from a marine Antarctic bacterium.

## 1. Introduction

Cold- and salt-tolerant enzymes are produced by extremophilic microorganisms adapted to grow under hostile environmental conditions. Cold-adapted enzymes, produced by psychrophilic or psychrotolerant microorganisms, generally show a high specific activity and remarkable structural flexibility at low temperatures. Their use as biocatalysts in biotech-based processes (such as manufacturing of food and beverage, detergents, pharmaceuticals, textile, leather, paper production processes and wastewater treatment and bioremediation) allows cost savings due the lack of heating and/or temperature stabilization procedures, it contributes to preserve thermolabile process components and it reduces the risk of contaminations (Marx et al., 2007). Salt-tolerant enzymes, produced by moderate and extreme halophiles (growing, respectively, at 3–15% or 15–30% w/v NaCl concentration) often maintain their activity at high ionic strength, in the presence of organic solvents and detergents, so that their use might reduce the need for pre-treatments and washing steps (De Lourdes Moreno et al., 2013; Yin et al., 2015).

**Figure 1:**
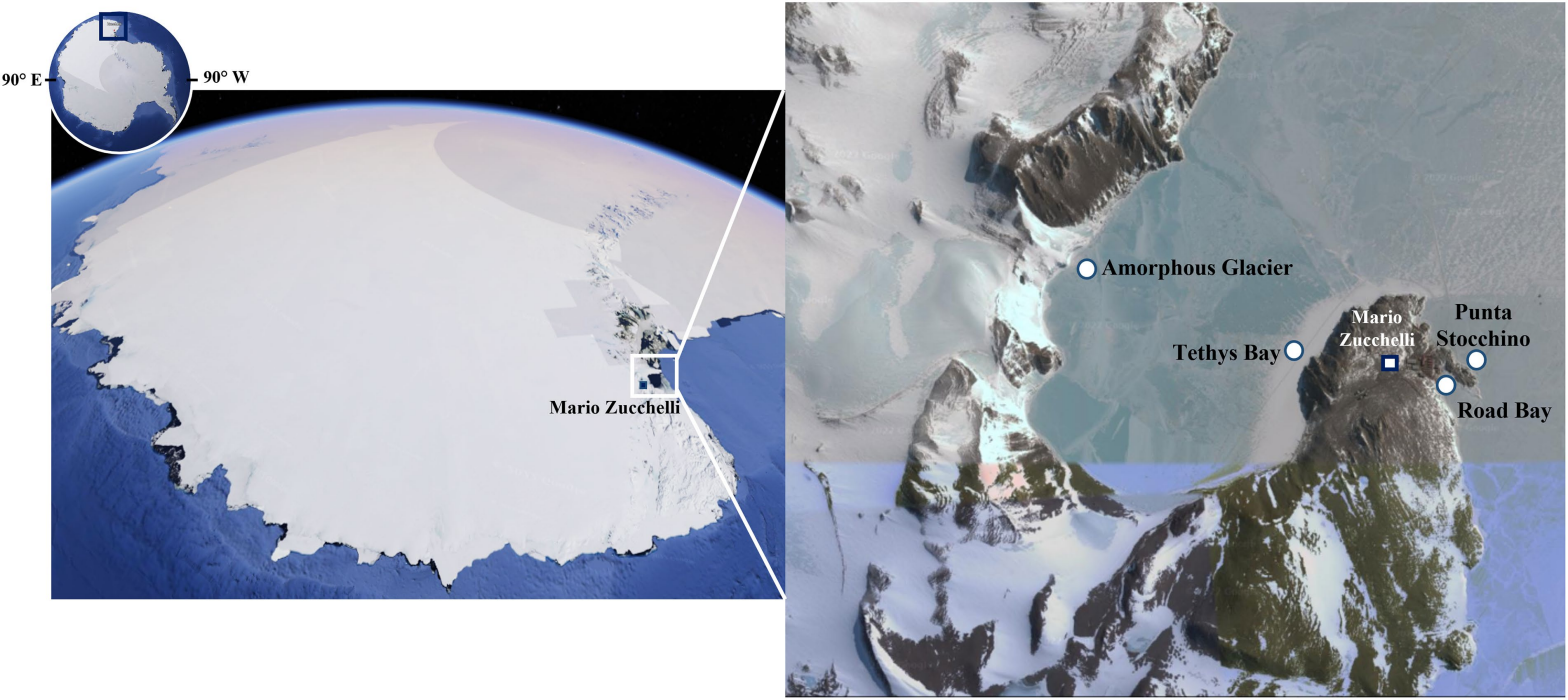
Map of the sampling sites in Terra Nova Bay (Antarctica). Modified from Google Earth (©2023, Google. U.S. Geological Survey. Data SIO, NOAA, U.S. Navy, NGA, GEBCO. PCG/NASA: NASA Landsat/Copernicus. https://www.google.it/intl/it/earth/). Within Road Bay area, the station Road Bay was affected by the wastewaters of the Zucchelli Station, while Punta Stocchino was a clean control site without anthropic impact. Within the Tethys Bay area, the station Amorphous Glacier was a site exposed to salinity gradient due to climate warming, while Tethys Bay station represented the control site, not exposed to natural perturbation.

Antarctica is known as the coldest, driest, windiest, and most inaccessible continent on Earth (Duarte et al., 2017). The survival and growth of organisms in this region is severely restricted by the high seasonable variability, with extremely low temperatures and light exposure in winter, and by the scarce availability of water and the associated osmotic stress. In spite of these conditions, microorganisms (mainly prokaryotes, but also fungi, microalgae, and protists) are able to colonize all Antarctic habitats, including lakes, rivers, ponds, streams, glaciers, rocks, and soils (Taton et al., 2003; Biondi et al., 2008; Brunati et al., 2009; Rojas et al., 2009; Zucconi et al., 2020). Thus, it is not surprising that in the last two decades, the number of studies exploring Antarctica microbial biodiversity as an untapped source of extremozymes has steadily grown (Núñez-Montero and Barrientos, 2018). Most of these studies focus on the microbiota of Antarctic soils or lakes (Obbels et al., 2016; Tytgat et al., 2016; Pessi et al., 2018). Indeed, little is still known about the psychrophilic and halophilic microbial communities living in the Antarctic Ocean, despite its wide extension and its biological species richness, fully comparable to that of temperate oceans (Marx et al., 2007). During the Italian National Antarctic Research Program ANT-biofilm (PNRA16_00105) project, marine microbial biofilms and seawater samples were collected from the Terra Nova Bay littoral (Ross Sea, Victoria Land), where the Italian research station Mario Zucchelli is located (Figure 1). From these samples, a total of 186 morphologically diverse microorganisms were isolated, by combining different isolation methods and using more than a dozen of cultivation media incubated at different temperatures. This yielded an Antarctic marine microbial strain collection, which could represent a promising source for novel extremozymes identification. In this paper we report on the screening of this collection to discover novel extremophilic laccases (EC 1.10.3.2, benzenediol: oxygen oxidoreductase).

Laccases are copper containing oxidases, mainly produced by fungi, bacteria, plants, and insects (Kumar and Chandra, 2020; Malhotra and Suman, 2021). They are able to catalyze oxidative coupling or bond cleavage of target compounds through four single-electron oxidation and radical formation, using oxygen as the final electron acceptor and reducing it to water. These enzymes show a wide substrate range, which is further broadened by the use of synthetic or natural redox mediators, such as 2,2′-azino-bis(3-ethylbenzothiazoline-6-sulfonic acid (ABTS) or syringaldehyde (Pollegioni et al., 2015; Chauhan et al., 2017; Yang et al., 2017). Furthermore, the 2,2′,6,6′-tetramethylpiperidine-N-oxyl (TEMPO)/laccase catalytic system has been proved to be an efficient chemo-enzymatic system for the oxidation of various classes of organic compounds under aerobic conditions, including benzyl alcohols and diols, both in sequential multi-step and cascade processes (Fabbrini et al., 2001; Díaz-Rodríguez et al., 2012; Brenna et al., 2017, 2018). Laccases are enzymes with powerful applications in a wide variety of industrial processes, such as (i) bleaching in the textile and dye industry, (ii) baking, juice processing and wine and beer stabilization in the food industry, and (iii) paper and fiberboards manufacturing in forest-product industry, where lignin removal or polymerization is required (Canas and Camarero, 2010; Pollegioni et al., 2015; Tonin et al., 2016; Mayolo-Deloisa et al., 2020). Laccases contribute to lignin utilization, being considered promising in converting plant biomass in integrated lignocellulose biorefineries for biofuel production (Roth and Spiess, 2015; Liu et al., 2020). Additionally, they can be applied in wastewater treatment and bioremediation for their ability to effectively degrade and detoxify various persistent organic pollutants, including dyestuffs, polycyclic aromatic hydrocarbons, pesticides, antibiotics, and other pharmaceuticals. They also find application in organic synthesis for the enzymatic conversion of chemical intermediates and in the production of pigments and antioxidants (Mayer and Staples, 2002; Mustafa et al., 2005; Alcalde, 2007; Yang et al., 2017). Laccases capable of tolerating extreme temperatures and pHs, salinity, and the presence of metals and chemical reagents are therefore required for such applications (Tonin et al., 2016; Duarte et al., 2017).

Apart from a previous report (Duarte et al., 2017), to our knowledge, the present study is one of the first contributes regarding the bioprospecting of the Antarctic microbial diversity for the screening and discovery of such novel promising extremophilic laccases.

## 2. Materials and methods

### 2.1. Sampling

During the “National Antarctic Research Program” (PNRA16_00105 – A1) in November 2017, artificial structures in stainless steel mounting plastic (polyvinyl chloride, PVC and polyethylene, PE) panels were deployed, through holes in the pack-ice, at −5 and − 20 meters (m) at four different stations of Terra Nova Bay: Road Bay (RB, Lat 74° 41.473’ S, Long 164° 07.125′E), Punta Stocchino (PTS, Lat 74° 41.651’S Long 164° 07.303′E), Tethys Bay (TB, Lat 74° 41.417’S Long 164° 06.303′E), and Amorphous Glacier (AG, Lat 74° 41.234’S Long 164° 02.135′E; Figure 1). Microbial biofilms growing on the panels and samples of surrounding seawater were collected three and 12  months after immersion (Caruso, 2020). Contextually to the sampling, the main physical–chemical parameters of the water (temperature, salinity, dissolved oxygen, pH, and fluorescence) were recorded at each station using a SeaBird 9/11 plus multiparametric probe (Caroppo et al., 2022). At the Zucchelli laboratory^1^, microbial biofilms were scraped using a sterile scalpel taking care to avoid contamination. They were in part serially diluted in sterile marine water and inoculated (0.1 mL) on Marine Agar plates (Conda Laboratories, Madrid, Spain), incubated at 4–5°C for 15 days minimum for culturable heterotrophic bacteria (Cappello et al., 2021), and in part stored at −20°C for shipment to and further analyses in microbiological labs in Italy (see below). Seawater samples received the same treatment above, i.e., plating on Marine Agar or storage and delivery to microbiology labs in Italy.

### 2.2. Microbial strains isolation from biofilms and seawater

At the arrival in Italy, 2.4 g of frozen biofilm samples were defrosted at 5°C, resuspended in sterile physiological solution (0.9% w/v NaCl) at a concentration of 2 mL per gram of biofilm and inoculated, either directly or serially diluted (10^−3^, 10^−6^ and 10^−9^), on 16 selective solid culture media (specific media and relative composition are reported in Supplementary Table SA). Before plating, part of the resuspended samples was subjected to a heat pre-treatment of 5 min at 55°C to enrich in spore-forming microorganisms. All media were added with nalidixic acid (25 mg/L; Merck KGaA, Darmstadt, Germany) and cycloheximide (50 mg/L; Merck KGaA) as selective agents to inhibit fast-growing Gram-negative bacteria and fungi, respectively. All the colonies isolated from biofilms and seawater samples plated on Marine Agar plates at Zucchelli station (see above) were also streaked in the above reported conditions.

To test the effect of temperature on microbial isolates, isolation plates were incubated at three temperatures: 4, 20 and 28°C for 1 month. Colonies were morphologically dereplicated, i.e., only one isolate was selected among those showing similar morphology considering color, size, shape, consistency and sporification observed at the stereomicroscope (Bel Photonics). Microscopic observation (Zeiss Primo Star, 40x magnification) followed to distinguish unicellular cocci or rods from filamentous microbes, multicellular structures, spores etc. The strains were also briefly phenotypically characterized according to the standard procedures in use at the CNR-ISP laboratory (Messina, Italy), including Gram staining, oxidase production, colony pigmentation, glucose fermentation and production of proteolytic, glycolytic and phosphatase enzymes (for detailed protocols, see Caruso et al., 2022; Supplementary Materials).

### 2.3. Growth conditions in cultivation broths

Microbial isolates were also tested for growth in liquid cultures. All liquid cultivation media were always added with 2% w/v artificial sea salt (Haquoss, Aquarialand, Torino, Italy), unless otherwise stated. Working Cell Banks (WCBs) were prepared by suspending microbial cells either in Luria Bertani (LB) broth, added with sterile glycerol at a final concentration of 15% v/v (unicellular bacteria), or in Tryptic Soy Broth (TSB; Conda Laboratories; filamentous actinomycetes) or in Shallow Stationary Culture medium (SSC) (filamentous fungi). SSC in g/L: 10 glucose, 0.198 ammonium tartrate, 2 KH_2_PO_4_, 0.5 MgSO_4_·7 H_2_O, 0.1 CaCl_2_·2 H_2_O, 1 thiamine, added with 1 mL/L trace elements solution after sterilization [in g/L: 15 sodium nitro-acetate, 3 MgSO_4_·7 H_2_O, 0.5 MnSO_4_·H_2_O, 0.1 NaCl, 0.1 FeSO_4_·7 H_2_O, 0.05 CuSO_4_·5 H_2_O, 0.1 CoSO_4_, 0.082 CaCl_2_·2 H_2_O, 0.1 ZnSO_4_·7 H_2_O, 0.01 AlK(SO_4_)_2_·12 H_2_O, 0.01 H_2_BO_3_, 0.01 NaMoO_4_] (Hong et al., 2016). WCBs were stored at −80°C. For unicellular bacteria, 0.1 mL of the WCB was inoculated in 10 mL LB broth in 50-mL sterile tubes, incubated at 20°C and under agitation at 180 revolutions per minute (rpm) for 24 h. 1 mL of the enrichment culture (1% inoculum) was transferred into 500-mL baffled Erlenmeyer flasks containing 100 mL of LB broth and incubated at 20°C and 180 rpm for 24 h. In the case of filamentous actinomycetes, 0.5 mL of the WCB was inoculated in 20 mL TSB in 100-mL Erlenmeyer flasks, incubated at 20°C and 180 rpm for 72 h. 5 mL of enrichment culture (5% inoculum) was transferred into 500-mL baffled Erlenmeyer flasks containing 100 mL of TSB, incubated at 20°C and 180 rpm for 120–144 h. For fungi, 0.5 mL of the WCB was inoculated in 20 mL SSC in 100-mL Erlenmeyer flasks, incubated at 20°C and 180 rpm for 96 h. 5 mL of enrichment culture (5% inoculum) was transferred into 500-mL baffled Erlenmeyer flasks containing 100 mL of SSC, incubated at 20°C and 180 rpm for 168–192 h.

### 2.4. Screening for laccase activities

As primary screening, isolates were grown on Mannitol Agar Medium (MAM) (in g/L: 20 mannitol, 2 KNO_3_, 2 MgSO_4_·7 H_2_O, 2 Na_2_HPO_4_, 15 agar) added with 2% w/v artificial sea salt and supplemented with ABTS (5 mM; Merck KGaA) or with azure B (25 mg/L; Merck KGaA). The plates were incubated at 20°C for 2 weeks. Colonies were selected as positive hits by the shifting toward green of plates color in the ABTS assay and by the appearance of a clear degradation halo in azure B-added agar. Secondary screening was conducted on microbial cultures grown in liquid (100 mL) as described above, after separation by centrifugation at 3,220 *g* for 30 min at 4°C of the biomass (pellet) from the supernatants. Enzyme activity assay was performed on both the supernatant of all the positive strains selected from the primary screening, and on their crude extracts prepared by sonication for 8 cycles of 30 s each on ice, followed by centrifugation at 38,724 *g* for 45 min at 4°C. Enzymatic activities, assayed using 0.05 mL samples from intracellular and extracellular fractions, were determined in 96-well plates, by using the automated liquid handling system epMotion 5075 (Eppendorf, Hamburg, Germany), on the following substrates (purchased from Merck KGaA): 1 mM ABTS (420 nm, ε_420nm_ = 36.0 mM^−1^ cm^−1^), 1 mM 2,6-dimethoxy phenol (2,6-DMP, 468 nm, ε_468nm_ = 49.6 mM^−1^ cm^−1^), or 10 mM 1,2-dihydroxy benzene (catechol, 410 nm, ε_410nm_ = 22.6 mM^−1^ cm^−1^) in 50 mM sodium acetate, pH 5.0, and 0.05 mM azure B (at 600 nm), 0.05 mM Remazol Brilliant Blue R (RBBR, at 600 nm), or 0.05 mM Reactive Black 5 (RB5, at 600 nm) in 50 mM sodium malonate, pH 4.5, added of 0.1 mM H_2_O_2_. The 96-well plates were incubated under shaking at 20°C for different times (5 min, 1, 5 and 24 h) and the absorbance at the specific wavelengths was recorded using a microtiter plate reader (Sunrise, Tecan, Switzerland). Controls with only the substrate and the corresponding buffer as negative controls were included. Each sample was evaluated in triplicate. The samples whose activity exceeded the control value were selected and the enzymatic activities were assayed spectrophotometrically at 20°C by monitoring 5 mM ABTS oxidation in 50 mM sodium acetate, pH 5.0 at 420 nm for 5 min.

Enzymatic activity on ABTS, 2,6-DMP and catechol was expressed as mU/mL, using the following equation:


1 $$\frac{\mathrm{mU}}{\mathrm{mL}}=\frac{\mathrm{\Delta Abs}/\min}{\varepsilon }\times \frac{V_{r}}{V_{s}}\times 1000$$

where one laccase activity unit (U) is defined as the amount of enzyme required to oxidize 1 μmol of ABTS, 2,6-DMP or catechol per min at 20°C, ∆Abs/min is the registered absorbance increase per minute, ε is the molar extinction coefficient of the substrate, and *Vr* and *Vs* are the total reaction volume and the volume of sample (enzyme) used, respectively. The values were normalized to mU/mg protein, using the total protein concentration of the samples quantified through the Biuret method (Gornall et al., 1949). Decolorization activities on azure B, RBBR and RB5 were expressed as percentage of decolorization, using the following equation:


2 $$\mathrm{Decolorization}\;\left(\%\right)=\left[\frac{\left(\mathrm{Ab}s_{t}-\mathrm{Ab}s_{i}\right)}{\mathrm{Ab}s_{i}}\right]\times 100$$

where Abs_t_ is the absorbance read at a given time, while Abs_i_ is the initial basal absorbance of the sample.

### 2.5. Growth and characterization of M68 bacterial isolate

M68 was isolated from Marine Agar plates prepared by inoculating a seawater sample collected from station RB at −20 m and phenotypically characterized following the methods described in (Caruso et al., 2022). Salt tolerance of M68 strain was tested either in liquid or in solid culture. 1% of enriched cultures was transferred into 500-mL Erlenmeyer flasks containing 100-mL of LB broth added with increasing NaCl concentrations (from 340 to 5,130 mM), and then incubated at 20°C and under agitation at 180 rpm for 24–48 h. The strain was considered tolerant to the salt concentration when the OD_600_ value reached at least 0.3 in 48 h (in comparison to a sample grown in standard LB broth). Meanwhile, the growth optimum for NaCl was defined in liquid medium comparing the 48 h growth in absence and in presence of different concentrations of salt. Growth curves at different temperatures (4, 20 and 30°C) were recorded as by OD_600nm_ detection; the optimal temperature was assumed to be when the highest OD_600_ increase was observed. In parallel, M68 strain was grown on LB agar plates added with increasing NaCl concentrations (from 340 to 5,130 mM), and incubated at different temperatures for 24–48 h. Growth was considered as positive when colonies become visible on the culture media within a time interval of 48 h.

### 2.6. DNA extraction and 16S sequencing of the bacterial isolate M68

M68 strain was grown in LB broth added with 2% w/v artificial sea salt: cells were harvested by centrifugation at 13,030 g for 15 min at 4°C and 500 mg pellet was first washed with 10.3% w/v saccharose and, after centrifugation, resuspended in 0.45 mL of TE buffer (50 mM Tris–HCl, pH 8.0 and 20 mM EDTA), added with 0.007 mL of RNAse (100 mg/mL). Following incubation at 37°C for 30 min, 0.05 mL of 5 M NaCl and 0.12 mL of 10% w/v SDS were added. The samples were incubated at 65°C for 80 min, then following 0.24 mL of 5 M potassium acetate addition, they were incubated at −20°C for 10 min. After centrifugation at 13,030 *g* for 15 min at room temperature (RT), 0.6 mL of supernatant were collected and mixed with 0.75 mL of 100% 2-propanol. The DNA pellet obtained by centrifugation at 13,030 *g* for 15 min at RT was washed in 70% v/v ethanol, air-dried, and finally resuspended in sterile deionized water. Genomic DNA extraction was verified by electrophoresis on 0.8% w/v agarose gel with Atlas ClearSight DNA Stain (Bioatlas, Tartu, Estonia). Molecular markers were from SibEnzyme (Novosibirsk, Russia). Genomic DNA concentration and its purity were determined at NanoDrop® UV–Vis spectrophotometer (ND-1000, Thermo Fisher Scientific, Waltham, Massachusetts, United States) and samples were kept at −20°C. 16S rDNA was sequenced by BMR Genomics (Padova, Italy) and sequence was deposited at GenBank (accession number OP804337).

### 2.7. Crude extract preparation from M68 strain

M68 bacterial strain was grown in 500-mL Erlenmeyer flasks containing 100 mL LB broth added with 2% w/v artificial sea salt and 2 mM CuSO_4_ (final concentration). Cells were collected after 24 h by centrifugation at 23,419 *g* for 45 min at 4°C. Cell extracts were obtained by sonication for 8 cycles of 30 s each on ice, followed by centrifugation at 38,724 *g* for 45 min at 4°C. The obtained supernatant was added with 30% w/v - corresponding to 176  g/L - (NH_4_)_2_SO_4_, and centrifuged at 38,724 *g* for 45 min at 4°C. The supernatant obtained after precipitation (SNP30) was used for the following kinetic studies.

### 2.8. Kinetic properties of the Ant laccase

The kinetic parameters were determined at 20°C in the presence of different concentrations of ABTS (from 0.125 to 10 mM) or 2,6-DMP (from 0.5 to 30 mM) in 50 mM sodium acetate, pH 5.0. The specific activity was expressed as mU per mg of total proteins. The kinetic data were fitted to the classical Michaelis–Menten equation.

Temperature dependence of enzymatic activity was determined by measuring 5 mM ABTS oxidation at 20°C in 50 mM sodium acetate, pH 5.0 in a 10–80°C temperature range, after pre-incubating buffer and substrate for 45 min at the selected temperature values. Enzyme preparation stability was measured at −20, 4, 20 and 37°C by incubating samples for 24 h and then determining the residual activity on 5 mM ABTS in 50 mM sodium acetate, pH 5.0 at 20°C.

pH dependence of enzymatic activity was determined by measuring 5 mM ABTS oxidation at 20°C in 12 mM multicomponent buffer (15 mM H_3_PO_4_, 15 mM Tris, 15 mM Na_2_CO_3_, 250 mM KCl), in the 3.0–9.0 pH range (Harris et al., 2001). Enzymatic activity was also assayed in 50 mM citrate–phosphate buffer, pH 5.0. The enzymatic stability at different pH values, was evaluated by incubating samples for up to 24 h in 50 mM sodium acetate, pH 5.0 or in 12 mM multicomponent buffer, pH 3.0, 4.0 and 5.0 at 20°C, and then determining the residual activity at different times on 5 mM ABTS in 50 mM sodium acetate, pH 5.0 at 20°C.

The effects of NaCl (0–1,000 mM), DMSO (0–40%) or Tween-80 (0–10%) on the enzymatic activity were assessed on 1 mM or 5 mM ABTS in 50 mM sodium acetate, pH 5.0 at 20°C.

Experiments were performed in triplicate and data were analyzed for statistical significance using one-way ANOVA followed by a Tukey’s multiple comparison test using GraphPad Prism software (GraphPad Software Inc., La Jolla, CA). Significance was assessed at *p* < 0.05.

### 2.9. Ant laccase identification by tandem mass spectrometry

Native-PAGE analysis was performed at 4°C on a 7.5% w/v acrylamide-resolving gel without SDS. Two different staining procedures were employed: (a) total protein composition was visualized by incubating the gel in fixing solution (40% v/v ethanol, 10% v/v acetic acid) for 30 min at RT and staining it with colloidal Coomassie staining solution (10% v/v orthophosphoric acid, 10% w/v ammonium sulphate, 0.12% w/v brilliant blue G, 20% v/v methanol) at 4°C overnight; (b) laccase activity was detected by incubating the gel at RT in 50 mM sodium acetate, pH 5.0, containing 10 mM ABTS. The protein band visualized by activity assay and the corresponding band on the Coomassie-stained gel were excised and conserved at 4°C.

The excised protein band from the Coomassie-stained gel was subjected to destaining, reduction, derivatization, and digestion with trypsin (1:10), and then analyzed with nLC-MS/MS. MS/MS analysis was carried out by an Orbitrap Fusion Tribrid mass spectrometer (Thermo Fisher Sci., Waltham, MA, United States), as previously described (Maffioli et al., 2020). Database search was performed using the Sequest search engine of Proteome Discoverer 1.4 (Thermo Fisher Sci.) against the proteome of *Halomonas* from Uniprot and NCBI sequence databases (released 12 December 2020). Only peptides with high Xcorr ≥ 1.5 and medium FDR confidence (FDR 0.05 relaxed) were kept for the identification. The minimum required peptide length was set to 6 amino acids with carbamidomethylation as fixed modification, Met oxidation and Arg/Gln deamidation as variable modifications. Multiple sequence alignment of the proteins identified through nLC-MS/MS was obtained using ClustalW in Uniprot and the Blastp tool (Align Sequences Protein BLAST) in NCBI (Eberini et al., 2002).

## 3. Results

### 3.1. Isolation of Antarctic microorganisms and screening for laccase activities

A total of 186 morphologically diverse microbes was isolated from the biofilm and seawater samples collected from the Terra Nova Bay. One hundred and forty-six microorganisms were isolated from biofilms grown on PVC and PE artificial panels deployed at the four sampling sites (RB, PTS, TB, and AG; Figure 1), and 40 from the surrounding seawater. The sampling campaign and the physical–chemical parameters of the sampling sites were recently described in Caroppo et al. (2022). Herein, samples were plated - in some cases after a heat pre-treatment to enrich in spore forming microorganisms and/or being serially diluted - on a variety of different isolation media (either with or without adding 2% w/v artificial sea salt) and incubated at three different temperatures, i.e., 4, 20 and 28°C, to capture as much as possible the cultivable diversity. All the selected microbes grew well on the media enriched with artificial sea salt, indicating that they are halotolerant, with only 28% of them strictly requiring salt addition to grow, being therefore considered obligate halophiles. For the microbial isolation, we used a salt concentration (2% w/v) that is slightly lower than that of the Antarctic Ocean, ranging from 3 to 3.5% w/v. This was done in order to account for the expected saline gradients that were present in the sampling area: at the RB site due to its proximity to the wastewater treatment plant of Mario Zucchelli research station and at the AG site, where the meltwater from the glacier is entering the sea (Caruso, 2020; Caroppo et al., 2022; Caruso et al., 2022). The majority of the isolates grew well at the three different temperatures used for plate incubation, showing that they tended to be psychrotolerant rather than psychrophilic. Only 8% grew better at 4°C in solid media, although their growth was detectable and generally abundant also at 20 and 28°C. By microscopical observation, 28 microorganisms were considered filamentous actinomycetes and 10 filamentous fungi (on the basis of the mycelium conformation, hyphae morphology and size, and in some cases spores’ presence), while the remaining ones generically resembled cocci and bacilli. Geographical distribution, sampling depth, sample origin and morphology-based classification of the 146 biofilm microbial isolates and of the 40 seawater microbial isolates are reported in Supplementary Figures S1, S2, respectively.

All the 186 microbial isolates were primarily screened for their ability to produce laccase enzymes on MAM plates (Casciello et al., 2017), added with 2% w/v artificial sea salt and supplemented with the soluble mediator ABTS (Yang et al., 2017) or with the dye azure B (Casciello et al., 2017). MAM was previously reported as an adequate medium for such screenings since it is known to support the growth of different types of microorganisms, without inducing pigment production, which can interfere with colorimetric enzyme assays (Casciello et al., 2017). After a 2 week-long incubation at 20°C, 25 microorganisms (19 unicellular bacteria, 1 filamentous actinomycete and 5 filamentous fungi) were able to oxidize ABTS, and 20 microorganisms (13 unicellular bacteria, 2 filamentous actinomycetes and 5 filamentous fungi) were able to degrade the dye azure B (Supplementary Table SB). Among them, 15 isolates showed both the activities on MAM plates. Geographical distribution, sampling depth, sample origin and morphology-based classification of these 30 microbial isolates are reported in Supplementary Figure S3.

Following this primary screening, the selected active strains were grown in liquid media (differently designed according to the morphology-based classification of the isolates, see Materials and Methods) to test their laccase-like activity production, by assaying either acellular broths (for putative secreted enzymes) or crude extracts after cell lysis by sonication (for intracellular enzymes). A broad range of substrates, i.e., ABTS, 2,6-DMP, catechol, azure B, RBBR and RB5, was evaluated. Twenty-five isolates confirmed their ability to oxidize ABTS, albeit at different extent; in addition, few of them converted 2,6-DMP (6 isolates) and catechol (4 isolates), and/or degraded azure B (12 and 5 isolates, respectively). The five additional isolates oxidizing only azure B in solid assay, confirmed this unique activity in liquid. None of the tested strains showed any enzymatic activity on RBBR and RB5 as substrates (Supplementary Table SB).

**Figure 2:**
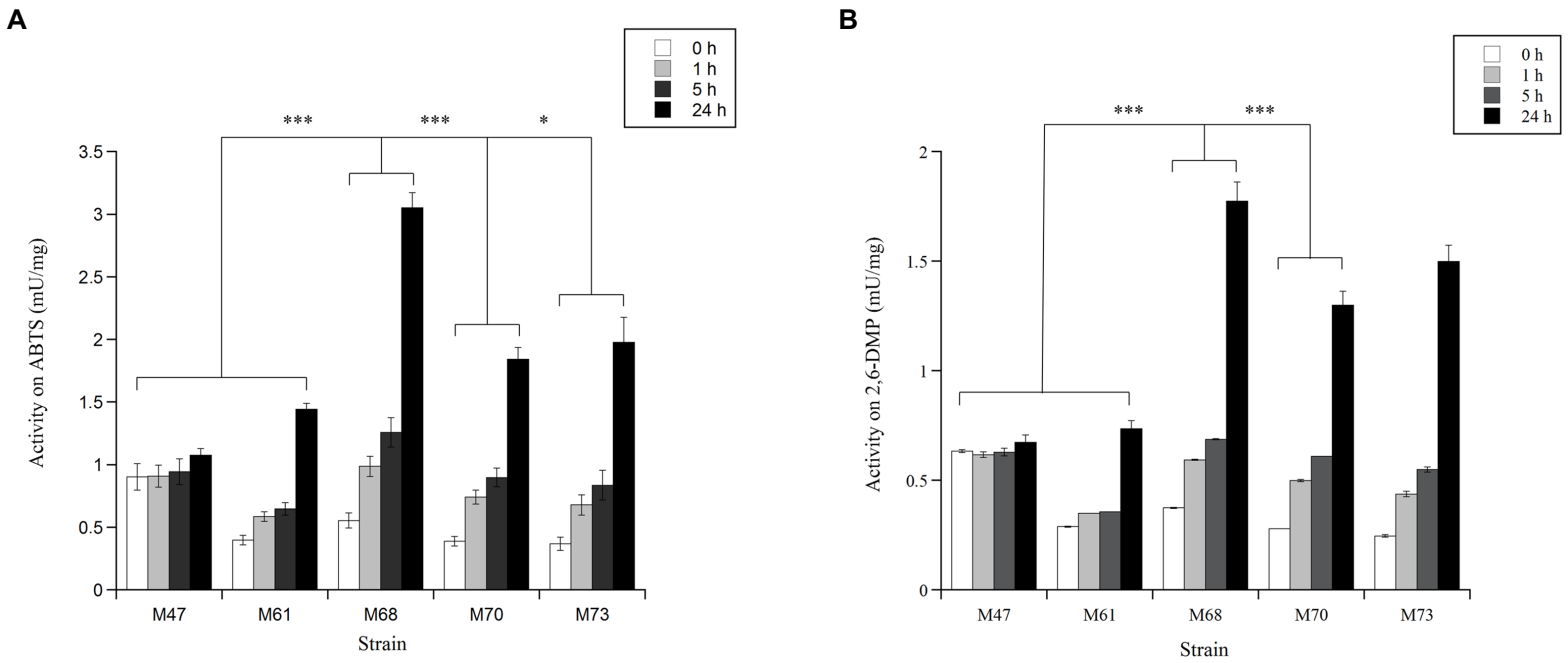
Laccase activity values detected in the crude extracts from the unicellular bacterial isolates M47, M61, M68, M70 and M73, assayed in 96-well plates at 20°C, on **(A)** 1  mM ABTS and **(B)** 1  mM 2,6-DMP, in 50  mM sodium acetate, pH 5.0. The absorbance was recorded immediately (t_0_) and 1, 5 and 24  h after substrate addition. The enzymatic activities are expressed as mU per mg of total proteins in the cellular extracts. Each sample was analysed in triplicate (mean ± standard deviation). The results were evaluated by statistical analysis using one-way ANOVA followed by a Tukey’s multiple comparison test. ***p < 0.0001, *p < 0.005.

The vast majority of these active microorganisms (21 isolates out of 30) originated from RB, which is the site located in front of the wastewater treatment plant discharge of the Mario Zucchelli station (Figure 1). Twenty-one were classified as cocci/bacilli on the basis of their microscopic morphology. In these unicellular bacteria, the laccase activity was prevalently detected intracellularly (Supplementary Table SB). The remaining active few actinomycetes (3) and fungi (6), instead secreted the oxidative enzymes, but their activity was low and poorly reproducible (Supplementary Table SB). Consequently, following our enzymatic activity-based screening approach, further investigations focused on the five most active isolates (M47, M61, M68, M70, and M73), which were unicellular bacteria isolated from seawater samples (Figures 2A,B). Almost all of these strains (M61, M68, M70, and M73) were isolated from RB samples, with the exception of M47 which was isolated from a TB sample. Details on a preliminary phenotypical characterization of the strains are reported in Supplementary Table SC. The intracellular enzyme preparations from these five selected isolates oxidized both ABTS and 2,6-DMP at 20°C, with a signal corresponding to the enzymatic activity being detected already after 1 h and increasing within the 24 h of incubation. For all the strains, the activity on ABTS was higher than on 2,6-DMP (Figures 2A,B). In this study, we focused on the laccase-activity produced by the M68 isolate, since it showed a significantly higher activity within the 24 h of incubation: 3.05 mU/mg of total proteins in the cellular extract on ABTS and 1.77 mU/mg on 2,6-DMP (Figures 2A,B). M68 did not show any activity either on catechol or azure B (Supplementary Table SB). The further investigation and in-depth taxonomical characterization of the other four positive strains will be considered in future works.

### 3.2. *Halomonas* sp. M68 characterization and Ant enzyme crude preparation

M68 strain was isolated from a seawater sample collected at −20 m in RB and it was found to be a Gram-negative, rod-shaped, low oxidase producing, glucose-sucrose-lactose not fermenting microorganism, producing orange pigmented colonies, with active proteolytic and phosphatase activities as assayed using fluorogenic substrates (Supplementary Table SC). This isolate grew without NaCl (both in solid and in liquid media) and tolerated up to 15% w/v (2.5 M) NaCl, with an optimal growth at 5% w/v (860 mM) NaCl. The sequencing of its 16S rDNA revealed that it belongs to the genus *Halomonas*, within the phylum *Proteobacteria* (γ-subclass). Specifically, a 16S rDNA sequence identity of 100% was found with two strains of *Halomonas meridiana* (strain PR51-13 and strain DSM 5425).

To further characterize the laccase-like intracellular enzyme activity detected in M68 strain (no activity was detected in the acellular broth), an optimization of the growing and production conditions was attempted by evaluating the enzyme activity at different growth temperatures (from 4 to 30°C), NaCl concentrations (from 340 to 5,130 mM) and cultivation times (4, 6, 8, 10, 12, and 24 h; Supplementary Figures S4A–C). Anyway, no significant improvement in enzyme activity was observed; thus, the original growing conditions were maintained (growth at 20°C at 2% w/v artificial sea salt for 24 h). 2 mM CuSO_4_ was also added to the cultivation broth, since Cu^2+^ is a cofactor of laccases (Pollegioni et al., 2015) and its addition to cultivation medium might increase laccase production (Ausec et al., 2017; Casciello et al., 2017; Berini et al., 2018). Under these culture conditions, the enzymatic activity of the cellular extract on 5 mM ABTS increased of about six-times up to 3 mU/mg of total proteins in the cellular extract (3.15 ± 0.38), compared to the activity detected in the absence of CuSO_4_ (0.55 mU/mg).

Following partial precipitation of the proteins in the cellular extract with ammonium sulphate at 30% w/v of saturation, the enzymatic activity was recovered in the supernatant (SNP30). Treatment at percentages of ammonium sulphate higher than 30% w/v led to enzyme inactivation. The total laccase activity of SNP30 (tested on 5 mM ABTS) increased from 265 to 670 mU and the specific activity reached a figure of 9.70 ± 0.96 mU/mg total proteins: the 2.5–3.1-fold increase in activity can result from both the removal of potentially interfering compounds or inhibitors and the positive effect of the increased ionic strength (see below). Notably, the laccase-like activity was not recovered after a dialysis step against 50 mM sodium acetate, pH 5.0, confirming the highest stability of the enzyme in the presence of salts. The SNP30 enzymatic preparation (that we start hereby to name Ant laccase) was therefore directly used for the kinetic studies.

### 3.3. Ant laccase identification through LC–MS/MS

Ant laccase was identified through nLC–MS/MS. SNP30 enzyme preparation was separated by electrophoresis in a native protein gel, which was then stained in parallel by a colloidal Coomassie stain (Supplementary Figure S5A) and a laccase activity-specific stain (Supplementary Figure S5B). In this last, the protein band responsible of laccase activity was identified and the corresponding band was excised from Coomassie-stained gel. nLC–MS/MS analyses on the band, briefly summarized in Table 1 and Supplementary Figure S6, clearly show that Ant laccase matches with three proteins correlated to a potential laccase-like activity: a copper resistance protein CopA from *H. meridiana* (ID A0A0D7UVK4, sequence coverage 30.64%), and two copper resistance system multicopper oxidases from two *Halomonas* species, i.e., *Halomonas piezotolerans* (ID WP_153843658.1, sequence coverage 24.38%) and *Halomonas* sp. FME66 (ID WP_193092891.1, sequence coverage 21.09%). Alignment of these three protein sequences using ClustalW from Uniprot database yielded a sequence identity of 96.7%, confirming that they belong to the same very well conserved family of the copper resistance system multicopper oxidase. This result was further confirmed by searching for similar or identical proteins in the NCBI *Halomonas* sequence database that allowed to identify at least 100 sequences with an identity ranging from 100 to 82.55%.

**Table 1:** List of the peptides identified by mass spectrometry. Mass spectrometry data were analysed by the Sequest search engine of Proteome Discoverer 1.4 (Thermo Fisher Sci.) against the proteome of *Halomonas* from Uniprot and NCBI sequence databases (released 12 December 2020). Only peptides with high Xcorr ≥ 1.5 and FDR confidence 0.05 were kept for the identification. The table reports the sequence of the peptides identified and their position in the protein, the XCorrelation (XCorr) value, the charge of the ion identified (Charge) and the monoisotopic molecular mass of the peptide ion (MH^+^).

Protein name	Sequence	XCorr	Charge	MH^+^
				[Da]
Copper resistance protein CopA [*Halomonas meridiana*]	44-TNVYAQGVEEGPEVSLAIR-62	4,59	2	2032,04
	64-ESLPIDGQEAQPITINGTSPAPLIR-88	4,30	2	2,617,38
	89-LKEGQDAVLR-98	2,97	2	1,128,64
	204-TMEGYYNFQER-214	3,05	2	1,437,61
	247-DIADVTGSTYTYLLNGHSPQENWNALFK-274	4,17	3	3,154,51
	283-VINGSAMSYFDVR-294	3,12	2	1,458,71
	301-MTVVAADGQPVQPVPVDEFR-320	5,04	2	2,155,09
	321-IGVAETYDVLVSPEDDRAYTIFAEAMDR-348	5,32	3	3,146,49
	441-IGANGLLLAGEAQPGSR-457	4,27	2	1,623,88
	514-FSEVTGPIHFVKDER-528	3,84	2	1760,89
Copper resistance system multicopper oxidase [*Halomonas piezotolerans*]	89-LKEGQDAVLR-98	2,97	2	1,128,64
	204-TMEGYYNFQER-214	3,05	2	1,437,61
	215-TIADFFADVR-224	2,66	2	1,154,58
	225-EKGFSQTAEMR-235	2,66	2	1,283,61
	247-DIADVTGSTYTYLLNGHSPQENWNALFK-274	4,17	3	3,154,51
	283-VINGSAMSYFDVR-294	3,12	2	1,458,71
	301-MTVVAADGQPVQPVPVDEFR-320	5,04	2	2,155,09
	321-IGVAETYDVLVSPEDDRAYTIFAEAMDR-348	5,32	3	3,146,49
	441-IGANGLLLAGEAQPGSR-457	4,27	2	1,623,88
Copper resistance system multicopper oxidase [*Halomonas sp. FME66*]	44-TNVYAQGVEEGPEVSLAIR-62	4,59	2	2032,04
	89-LKEGQDAVLR-98	2,97	2	1,128,64
	204-TMEGYYNFQER-214	3,05	2	1,437,61
	215-TIADFFADVR-224	2,66	2	1,154,58
	283-VINGSAMSFFDVR-294	2,75	2	1,458,71
	301-MTVVAADGQPVQPVPVDEFR-320	5,04	2	2,155,09
	321-IGVAETYDVLVSPEDDRAYTIFAEAMDR-348	5,32	3	3,146,49
	441-IGANGLLLAGEAQPGSR-457	4,27	2	1,623,88

### 3.4. Kinetic properties of Ant laccase

**Figure 3:**
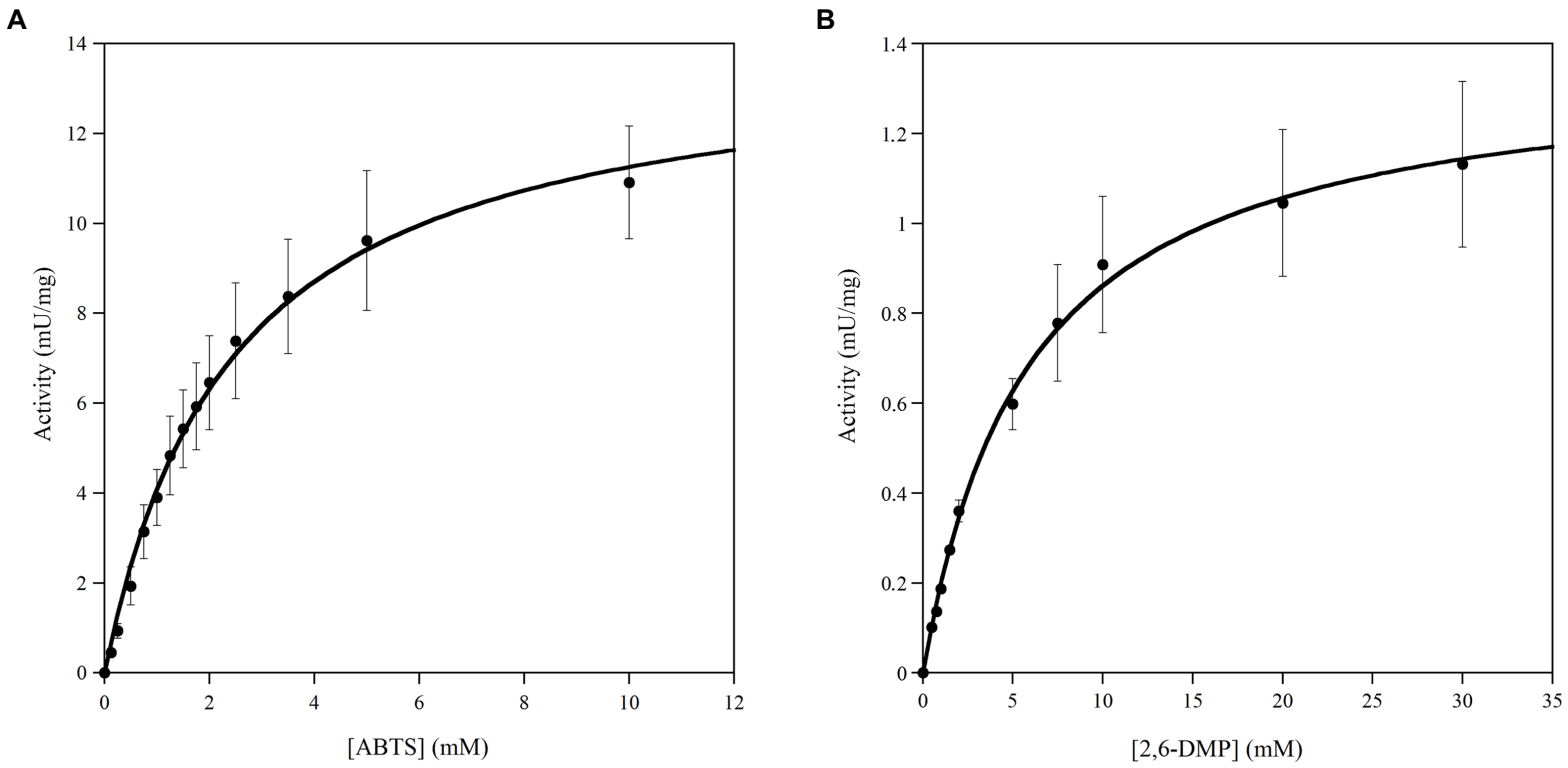
Michaelis–Menten plots of kinetic of Ant laccase preparation (SNP30) on the substrates: **(A)** ABTS and **(B)** 2,6-DMP. All measurements were performed at 20°C in 50  mM sodium acetate, pH 5.0. Values represent the means of three independent experiments (mean ± standard deviation).

Kinetic parameters of Ant laccase preparation were determined on the non-phenolic ABTS and the phenolic 2,6-DMP as substrates. In all cases, the dependence of the activity values on the substrate concentration followed a Michaelis–Menten kinetic (Figures 3A,B). The highest maximal activity was observed on ABTS as substrate (13.99 ± 0.41 mU/mg protein) that also showed a lower K_m_ value (approx. 2 mM), while the activity determined on 2,6-DMP was *ca.* 10-times lower (1.37 ± 0.03 mU/mg protein). The enzymatic preparation did not possess any dye-decolorizing activity (i.e., on azure B, RBBR and RB5) and no activity was detected on catechol as substrate, confirming what observed during the primary and secondary screening (Supplementary Table SB).

### 3.5. Effect of temperature and pH on Ant laccase activity

**Figure 4:**
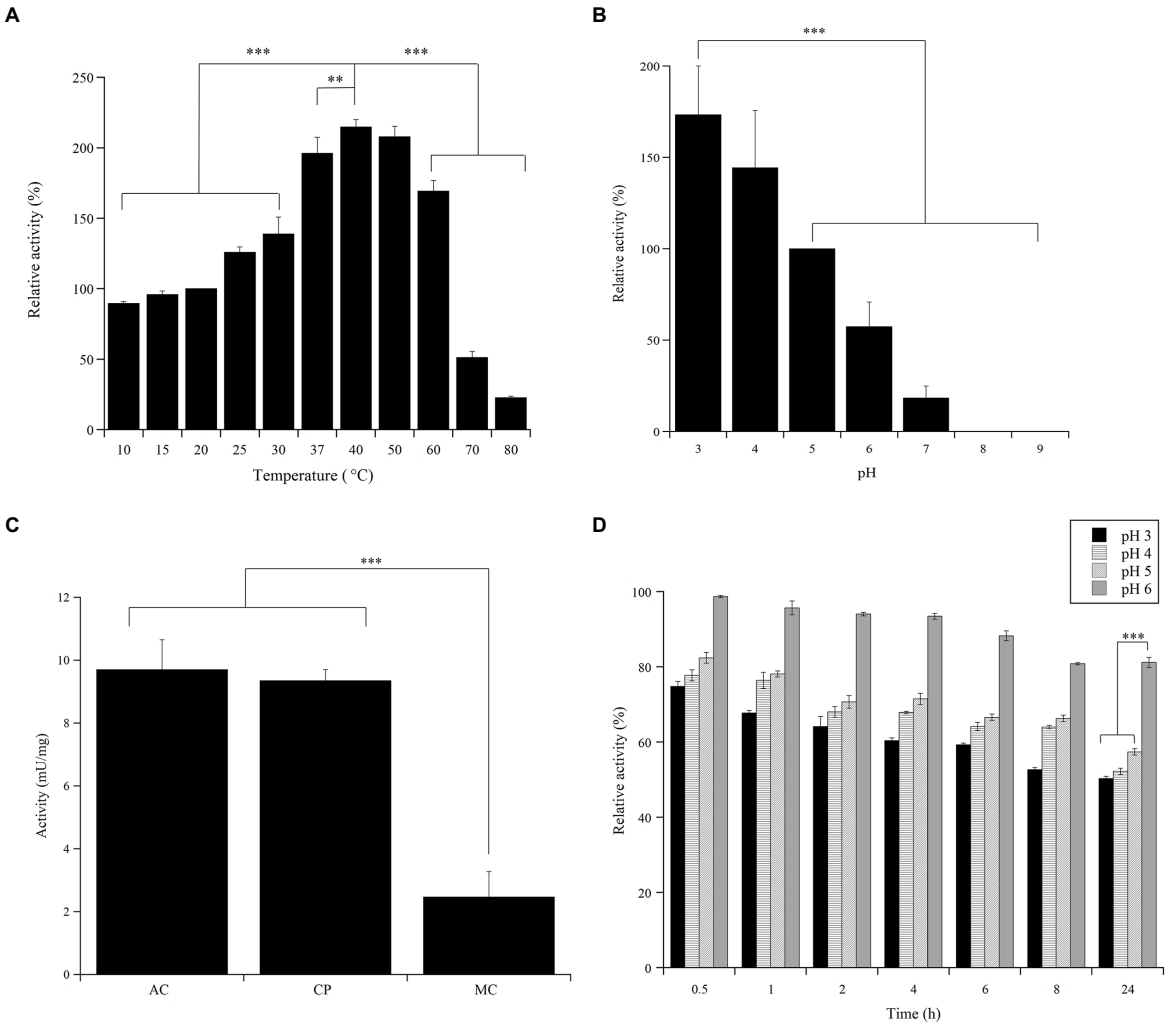
Effect of pH and temperature on the activity and stability of the Ant laccase preparation from *Halomonas* sp. M68 (SNP30). **(A)** Temperature effect on the enzyme activity assayed on 5  mM ABTS in 50  mM sodium acetate, pH 5.0. The value at 20°C was taken as 100%. **(B)** pH effect on the enzyme activity assayed on 5 mM ABTS in 12 mM multicomponent buffer and at 20°C. The value at pH 5.0 was taken as 100%. **(C)** Comparison of the specific activity (mU/mg protein) on 5  mM ABTS at 20°C in different buffers: 50  mM sodium acetate (AC), pH 5.0, 50  mM citrate–phosphate (CP), pH 5.0, 12  mM multicomponent (MC) buffer, pH 5.0. **(D)** pH effect on the stability of the enzyme preparation. The residual activity was measured on 5  mM ABTS in 50  mM sodium acetate, pH 5.0 during 24  h of incubation at 20°C in the various buffers. The activity value at time = 0 at each pH value was taken as 100%. Values represent the means of three independent experiments (mean ± standard deviation). The results were evaluated by statistical analysis using one-way ANOVA followed by a Tukey’s multiple comparison test. ***p < 0.0001, **p < 0.001.

The activity of the Ant laccase preparation (SNP30) from *Halomonas* sp. M68 on ABTS was determined at different temperature and pH values. The optimum temperature for enzyme activity was at around 40°C, with activity values increasing of more than two-fold compared to that at 20°C. Nevertheless, nearly 90% of the enzyme activity measured in standard conditions (20°C), and more than 40% of that measured at 40°C, were preserved even at 10°C (Figure 4A): the enzyme maintained at least 50% of its maximum activity in the 10–60°C temperature range. A significant decrease in enzyme activity was observed only at >60°C. The Ant laccase preparation was quite stable: no significant loss of activity was apparent after 24 h incubation at −20, 4 and 20°C, and when incubated for 24 h at 37°C, *ca.* 65% of its initial activity was maintained (Supplementary Figure S7).

The highest activity of the Ant enzyme preparation was detected at acidic pH values (Figure 4B), highlighting a strong dependence of the enzyme activity on buffer composition even at optimal pH (Figure 4C). Ant laccase activity strongly decreased at pH 7.0 (reaching approx. 20% of that measured in standard conditions, at pH 5.0) and the enzyme was completely inactive at pH >8.0. The preparation possessed a good stability in the 3.0–6.0 pH range following incubation for 24 h at 20°C, showing the highest residual activity at pH 5.0–6.0 (Figure 4D).

### 3.6. Effect of NaCl, solvents, and detergents on Ant laccase activity

**Figure 5:**
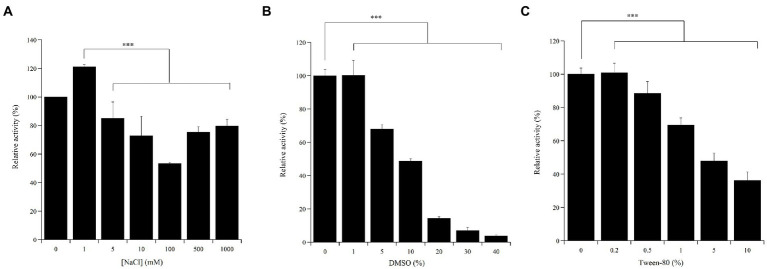
Effect of NaCl **(A)**, DMSO **(B)** and Tween-80 **(C)** concentrations on Ant laccase activity in the crude enzyme preparation from *Halomonas* sp. M68 (SNP30). Enzyme activity was measured on 1  mM **(A)** or 5  mM (**B, C**) ABTS in 50  mM sodium acetate pH 5.0. The value in absence of the different compounds was taken as 100%. Values represent the means of three independent experiments (mean ± standard deviation). The results were evaluated by statistical analysis using one-way ANOVA followed by a Tukey’s multiple comparison test. ***p < 0.0001.

High salt concentrations can affect the activity of laccases thus hampering various putative biotechnological applications. The Ant enzyme preparation well tolerated high NaCl concentrations, up to 1 M, while still maintaining *ca.* 80% of the activity measured in the absence of the sodium salt (Figure 5A). Additionally, to verify the potential for using the Ant laccase in processes requiring solvents and surfactants, the effects of DMSO and Tween-80 on its enzymatic activity were evaluated. Compared to the standard activity assayed in plain buffer, the activity of the enzyme preparation halved in the presence of 10% v/v DMSO or of 5% v/v Tween-80 (Figures 5B,C, respectively).

## 4. Discussion

In the search for novel robust laccases for industrial biotechnological applications, we screened our recently built Antarctic strain collection. This collection comprises 186 morphologically different microorganisms (mainly bacteria) isolated, by means of a combination of different approaches, from biofilms grown on PVC and PE panels and seawater samples collected at different depths at four sites along the Terra Nova Bay littoral (Ross Sea). The distribution of the positive strains for laccase-like activity selected during the primary screening (30 out of 186) indicated that most of them (21) originated from RB site, from which most of the isolates of the collection came from. This is the most impacted site among the four sampled in this Antarctic campaign due to its proximity to the discharge of the wastewater treatment plant of Mario Zucchelli station (Caruso et al., 2022; Caroppo et al., 2022). Twenty-one of the selected positive strains were classified at a morphological level as unicellular bacteria and they were isolated from marine seawater, while the remaining 9 were filamentous actinomycetes or filamentous fungi originated by biofilms growth on the plastic submerged supports. The percentage of the isolates positive for their activity on ABTS was 13.4, and 10.8% on azure B. To our knowledge, only one previous study reported on the screening of Antarctic microorganisms for laccase activities, but data are not comparable considering that it was related to 61 yeast strains isolated from soil samples collected in Mayo/King George Island (Antarctica): 33% of them showed significant activity for dye decolorization, 25% for laccase activity and 38% for ligninolytic activity (Rovati et al., 2013).

Among the 30 strains herein selected as putative laccase producers, in the secondary screening M68 showed the highest activity on ABTS, compared to the other strains. M68 was psychrotolerant and halotolerant. On the basis of 16S rDNA sequencing, it was attributed to the genus *Halomonas* (γ-Proteobacteria), with a 100% sequence identity to two strains of *H. meridiana* (strain PR51-13 and strain DSM 5425). *H. meridiana* DSM 5425 is the type-strain for the *H. meridiana* species and it was originally isolated during a limnological survey in 1988 from saline lakes of the Vestfold Hills region (Antarctica; James et al., 1990). Strain PR51-13 was identified as a member of a polycyclic aromatic hydrocarbon (PAH)-degrading bacterial consortia obtained through PAH enrichment from deep-sea water above the Southwest Indian ridge (Yuan et al., 2015). In fact, members of the *Halomonas* genus tend to be moderately to extremely halotolerant or halophiles and have been isolated from a variety of saline environments, such as marine water, hypersaline lakes, saline soils, meat-curing brines, and sewage treatment plants (Arahal et al., 2002). They have already been proposed as candidates for the production of different compounds of biotechnological and industrial relevance, such as polyhydroxyalkanoates, ectoines, biosurfactants, bioemulsifiers and various halotolerant hydrolases (Yin et al., 2015; Biswas et al., 2022). An extracellular α-amylase from *H. meridiana* was isolated and characterized by Coronado and co-authors, showing 90% of its maximal activity even at NaCl concentrations higher than 15% (Coronado et al., 2000). An azoreductase degrading Reactive Red 195 at 5–15% NaCl was also recently characterized from *H. meridiana* SAIBP-6 strain, isolated from marine sediments of Andaman Sea, India (Saha et al., 2022). Despite their potential, to our knowledge, no laccases have been identified and functionally characterized from these *Halomonas* spp. yet.

The Ant laccase that we identified in *Halomonas* sp. M68 showed the highest aminoacidic sequence coverage with a copper resistance protein CopA from *H. meridiana*, consistently with the identification of M68 as a member of the same species. CopA was reported to have more than one biological function, playing a role in both copper resistance and as catalyst in oxidative reactions (Cha and Cooksey, 1991; Mills et al., 1993). It accumulated intracellularly indicating that its main physiological role could be related to UV protection, pigmentation, sporulation, metal oxidation or copper homeostasis (Yang et al., 2017). The proposed mode of action of CopA was reported to involve a methionine rich region, which binds several atoms of copper (Bielli and Calabrese, 2002; Xiao and Wedd, 2011). Noteworthy, the production of Ant laccase is triggered by the culture medium addition of 2 mM CuSO_4_, leading to a 6-fold increase in enzyme activity. Cu^2+^ role in laccase active site is well-known as well as its modulation of laccase gene transcription and post-transcription modifications (D’Souza et al., 2006; Nakade et al., 2013; Yang et al., 2013; Passarini et al., 2015; Pollegioni et al., 2015; Theerachat et al., 2019).

Ant laccase preferential substrate was ABTS, and the highest activity was measured at acidic pH values (3.0–5.0), although it was mostly stable at pH ≥ 5.0, following a common behavior among laccases (Tonin et al., 2016). It was also active on 2,6-DMP as a substrate, while neither dye-decolorizing activity, nor oxidation of catechol was detected.

Concerning temperature, Ant laccase showed its maximum activity at around 40°C, which is lower than the optimal temperatures (70–80°C) frequently reported for laccases from both fungal and bacterial sources (Tonin et al., 2016). This feature might be expected considering that this enzyme is produced by a psychrotolerant marine Antarctic bacterium. Consistently, Ant laccase maintained at 10°C more than 40% of its maximal activity. This behavior hints at a cold-adaptation of Ant from an ancestral mesophilic precursor and this laccase could result particularly versatile and able to well adapt to sudden changes in temperatures of the surrounding environment. Similar thermostability results were previously reported only for a few laccases isolated from fungal strains (Wang et al., 2009; Tian et al., 2020), and in particular, for a laccase-like enzyme Lac1326 derived from a marine metagenomic library, whose optimum temperature for activity was found to be 60°C (Yang et al., 2018).

Ant laccase was also shown to be particularly stable, with 65% of its starting activity maintained even after 24 h incubation at 37°C: this could favor its use in bioremediation treatments in open environments, where there is no temperature control, or in the food industry when low to moderate heating is applied to preserve thermolabile components (Brijwani et al., 2010; Yang et al., 2017). Considering the role of oxygen in the laccase-catalyzed reaction (Fabbrini et al., 2001; Ortner et al., 2015; Brenna et al., 2018), the dependence on temperature of oxygen solubility in water, and the relevant applications of this oxidase, future investigations should compare the performances of Ant laccase and mesophilic intracellular laccases at different oxygen pressure. Another major obstacle in using laccases in municipal and industrial wastewater treatment, which are known to contain significant amounts of salts, is represented by their sensitivity to halide ions, which causes their rapid inactivation (Theerachat et al., 2019; Unuofin et al., 2019). The Ant laccase from *Halomonas* sp. M68 showed favorable halotolerant features for such industrial applications (Espina et al., 2021). Although it was not strongly activated by NaCl, as it has been reported for some laccases isolated from other marine derived strains (Wikke et al., 2019), in the presence of 1 M NaCl it maintained 80% of its activity detected in salt-free conditions. A possible explanation is that Ant, under physiological conditions, is partially protected towards saline variations, since it is produced as an intracellular enzyme. In fact, halophilic and halotolerant bacteria living in hypersaline environments tend to avoid letting salts diffuse into their cells, by accumulating intracellularly osmolytes and various compatible solutes, in order to balance the osmotic pressure (Yin et al., 2015). This phenomenon was also reported for two azoreductases isolated from *Halomonas* species, which were tolerant to NaCl but showed their highest activity in its absence (Eslami et al., 2016; Tian et al., 2019).

Finally, Ant laccase proved to tolerate well the presence of organic solvents, such as DMSO, one of the most active organic compounds used to solubilize organic macromolecules (including lignin), and of surfactants, such as Tween-80, which can be used in wastewater treatment and in lignin pretreatment prior to enzyme hydrolysis (Tonin et al., 2016). In fact, Ant laccase maintained approximately 50% of the activity detected in plain buffer at 10% DMSO or 5% Tween-80.

We can conclude that the various characteristics displayed by the *Halomonas* sp. M68 Ant laccase (i.e., cold-adaptation, and thermo-, halo- and solvent-tolerance) make it a promising candidate for biotechnological and industrial use. To the best of our knowledge, this is the first report on a thermo- and halo-tolerant laccase isolated from a marine Antarctic bacterium. Foreseeing Ant laccase possible developments, future studies should be devoted to favor its secretion by the homologous host or by a heterologous one. The extracellular localization of Ant laccase might in fact greatly facilitate protein recovery and its biotechnological use.

## Data availability statement

The data presented in the study are deposited in the GenBank (https://www.ncbi.nlm.nih.gov/genbank/) repository, accession number OP804337.

## Author contributions

MB, EB, and ER designed the study. GC, OD, and MA organized the expedition to Antarctica and collected samples. MB, LP, EB, and ER performed the experiments and wrote the draft of the manuscript. GT and EM performed mass spectrometry analysis. GC, LP, and FM coordinated the project and reviewed the manuscript. All authors contributed to the article and approved the submitted version.

## Funding

This research was funded by the PNRA16_00105 ANT-Biofilm Project (Microbial colonization of Antarctic benthic environments: response of microbial abundances, diversity, activities and larval settlement to natural and anthropogenic disturbances and search for secondary metabolites) and Consorzio Interuniversitario per le Biotecnologie (CIB), Bando MiUR Consorzi “Network-CIB: catalisi dell’innovazione nelle biotecnologie,” subproject: “Un approccio glocal alle bioraffinerie di terza generazione.”

## Conflict of interest

The authors declare that the research was conducted in the absence of any commercial or financial relationships that could be construed as a potential conflict of interest.

## Publisher’s note

All claims expressed in this article are solely those of the authors and do not necessarily represent those of their affiliated organizations, or those of the publisher, the editors and the reviewers. Any product that may be evaluated in this article, or claim that may be made by its manufacturer, is not guaranteed or endorsed by the publisher.

## Footnotes

^1^ https://www.isp.cnr.it/index.php/en/infrastructures/research-stations/mario-zucchelli
