# Supplementary material for: A novel promising laccase from the psychrotolerant and halotolerant Antarctic marine Halomonas sp. M68 strain
Source: Front Microbiol. 2023 Feb 10;14:1078382. doi: 10.3389/fmicb.2023.1078382 (PMC9950745; doi:10.3389/fmicb.2023.1078382)
Supplement: Supplementary file 1 [file Data_Sheet_1.docx]

Supplementary Material

**Supplementary Table A**

| **Medium** | **Composition (in g/L)** | **Reference** |
| --- | --- | --- |
| **Antarctic Bacterial Medium** | 5 bacteriological peptone, 2 yeast extract, 20 agar | Reddy et al., 2000 |
| **Chitin Agar (*)** | 4 colloidal chitin, 0.7 K_2_HPO_4_, 0.5 MgSO_4_·7 H_2_O, 0.3 KH_2_PO_4_, 0.01 FeSO_4_, 0.001 ZnSO_4_, 0.001 MnCl_2_, 20 agar  Colloidal chitin was prepared from chitin from shrimp shells (Merck KGaA, Darmstadt, Germany) according to the protocol of Hsu et al., 1975 | Hsu et al., 1975 |
| **Isolation Streptomyces Project agar (ISP) 2 (*)** | 10 dextrose, 5 bacteriological peptone, 3 yeast extract, 3 malt extract, 20 agar  (Himedia Laboratories, Mumbai, India) | Marcone et al., 2017 |
| **ISP4 (*)** | 10 soluble starch, 2 (NH_4_)_2_SO_4_, 2 CaCO_3_, 1 K_2_HPO_4,_ 1 MgSO_4_·7 H_2_O, 1 NaCl, 0.001 FeSO_4_·7 H_2_O, 0.001 MnCl_2_·7 H_2_O, 0.001 ZnSO_4_·7 H_2_O, 20 agar  (Himedia Laboratories, Mumbai, India) | Marcone et al., 2017 |
| **ISP5 (*)** | 1 L-asparagine, 1 K_2_HPO_4_, 10 mL/L glycerol, 1 mL/L Trace Elements Solution (0.001 FeSO_4_·7 H_2_O, 0.001 MnCl_2_·7 H_2_O, 0.001 ZnSO_4_·7 H_2_O), 20 agar  (Himedia Laboratories, Mumbai, India) | Marcone et al., 2017 |
| **ISP6 (*)** | 15 bacteriological peptone, 5 proteose peptone, 1 yeast extract, 1 K_2_HPO_4_, 0.5 ferric ammonium citrate, 0.08 sodium thiosulphate, 15 agar  (Himedia Laboratories, Mumbai, India) | Marcone et al., 2017 |
| **ISP7 (*)** | 1 L-asparagine, 0.5 L-tyrosine, 0.5 K_2_HPO_4_, 0.5 MgSO_4_·7 H_2_O, 0.5 NaCl, 15 mL/L glycerol, 1 mL/L Trace Elements Solution (in mg/L: 2.85 H_3_BO_3_, 1.8 MnCl_2_·4 H_2_O, 1.77 sodium tatrate, 1.36 FeSO_4_·7 H_2_O, 0.04 CoCl_2_·6 H_2_O, 0.027 CuCl_2_·2 H_2_O, 0.025 Na_2_MoO_4_·2 H_2_O, 0.02 ZnCl_2_), 20 agar  (Himedia Laboratories, Mumbai, India) | Marcone et al., 2017 |
| **Marine Agar** | 19.4 NaCl, 8.8 MgCl_2_, 5 bacteriological peptone, 3.24 Na_2_SO_4_, 1.8 CaCl_2_, 1 yeast extract, 0.55 KCl, 0.16 NaHCO_3_, 0.1 ferric citrate, 0.08 KBr, 0.022 H_3_BO_3_, 0.008 Na_2_HPO_4_ 0.004 sodium silicate, 0.0034 SrCl_2_, 0.0024 NaF, 0.0016 NH_4_NO_3_, 15 agar  (Conda Laboratories, Madrid, Spain) | Caruso et al., 2022 |
| **Oatmeal Agar** | 20 oatmeal, 20 agar | Marcone et al., 2017 |
| **Seawater Agar** | 20 Haquoss artificial sea salt, 15 agar | Baxter and Sieburth, 1984 |

**Supplementary Table A.** Solid media used in the heterotrophic marine bacteria isolation procedure from Antarctic seawater and biofilm samples. Composition (in g/L) and commercial suppliers are reported. (*) indicates media which were also added with 20 g/L (2% w/v) artificial sea salt (Haquoss, Aquarialand, Torino, Italy). All the media components, unless otherwise stated, were obtained from Merck KGaA, (Darmstadt, Germany).


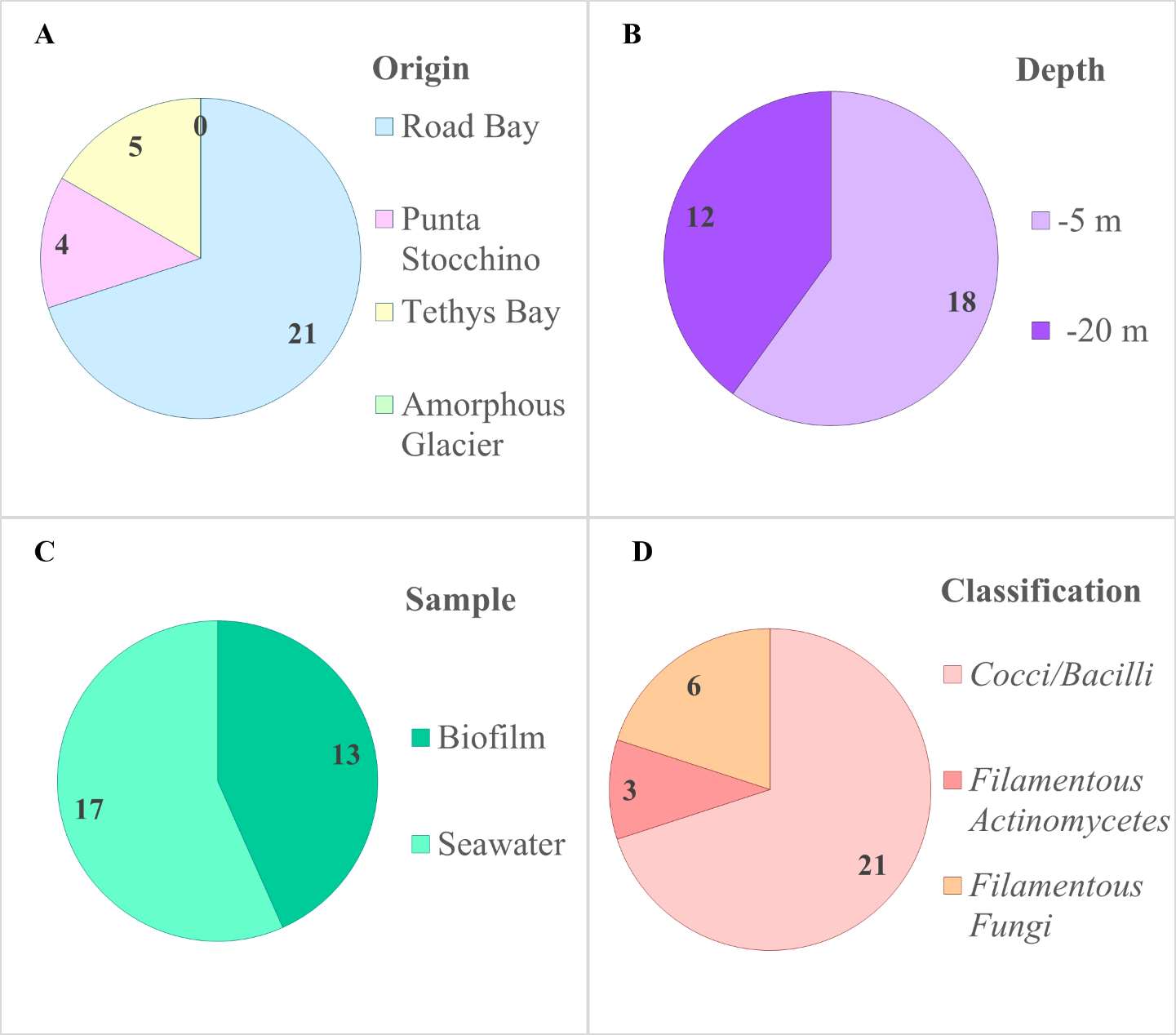

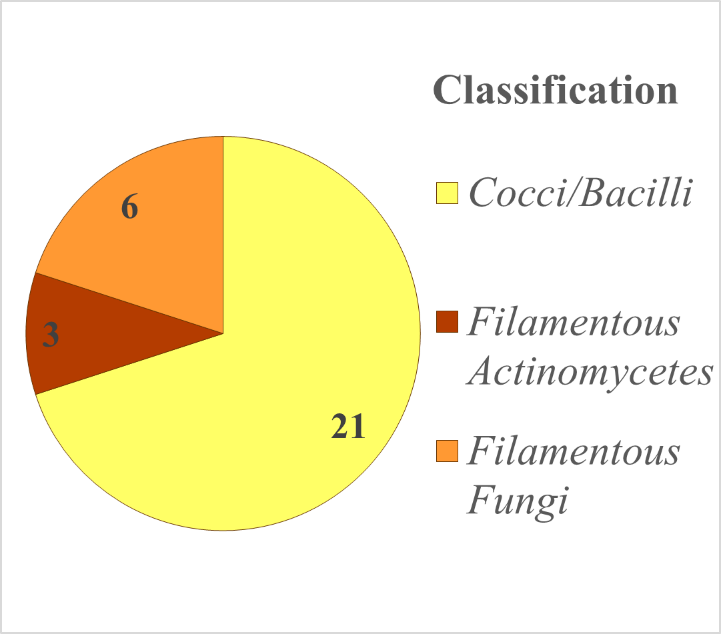


**D**

**Supplementary Figure 1.** Geographical distribution (A), sampling depth (B), plastic panel of origin (C) and morphology-based classification (after dereplication, see Material and Methods) (D) of the 146 microbial isolates from the Antarctic biofilms samples collected from polyvinyl chloride (PVC) or polyethylene (PE) panels at a depth of -5 or -20 m in the period from November 2017 to November 2018 from the four sites: Road Bay (RB), Punta Stocchino (PTS), Tethys Bay (TB) and Amorphous Glacier (AG).


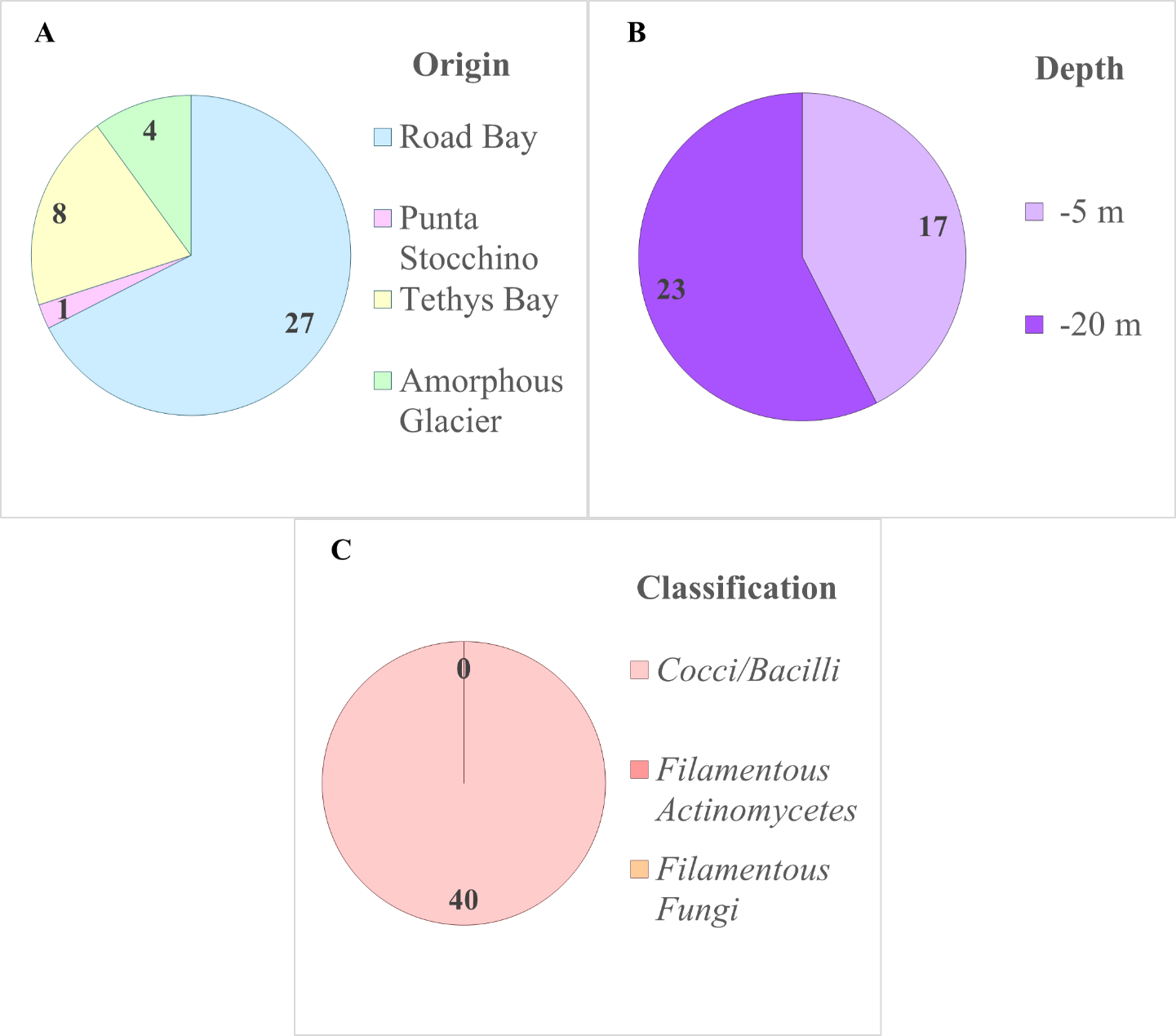

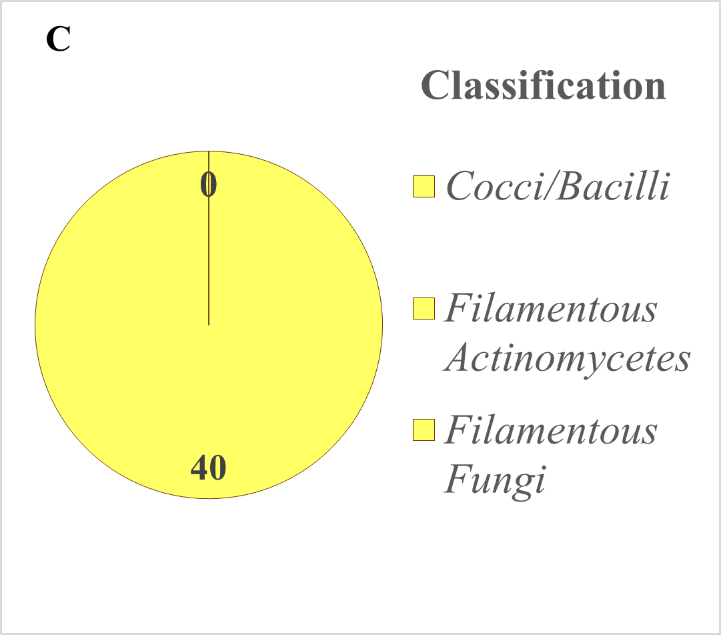

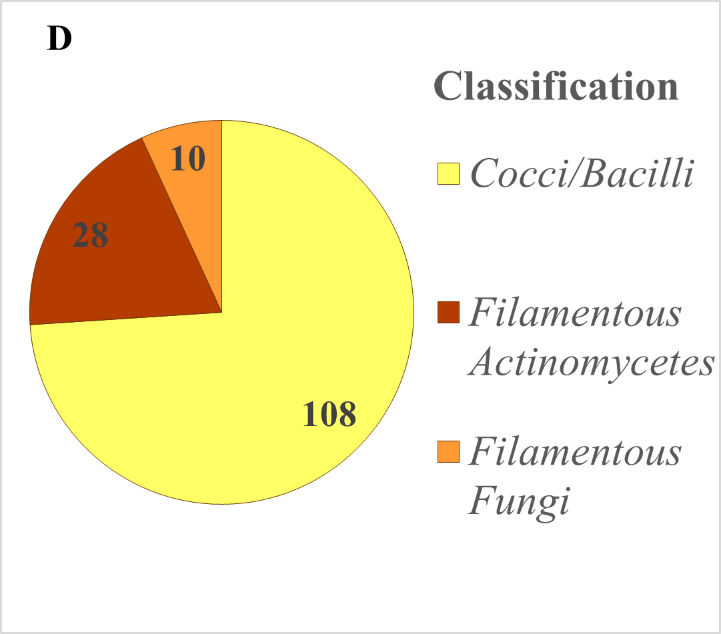


**Supplementary Figure 2.** Geographical distribution (A), sampling depth (B) and morphology-based classification (after dereplication, see Material and Methods) (C) of the 40 microbial isolates from the Antarctic seawater samples collected at a depth of -5 or -20 m in the period from November 2017 to November 2018 from the four sites: Road Bay (RB), Punta Stocchino (PTS), Tethys Bay (TB) and Amorphous Glacier (AG).


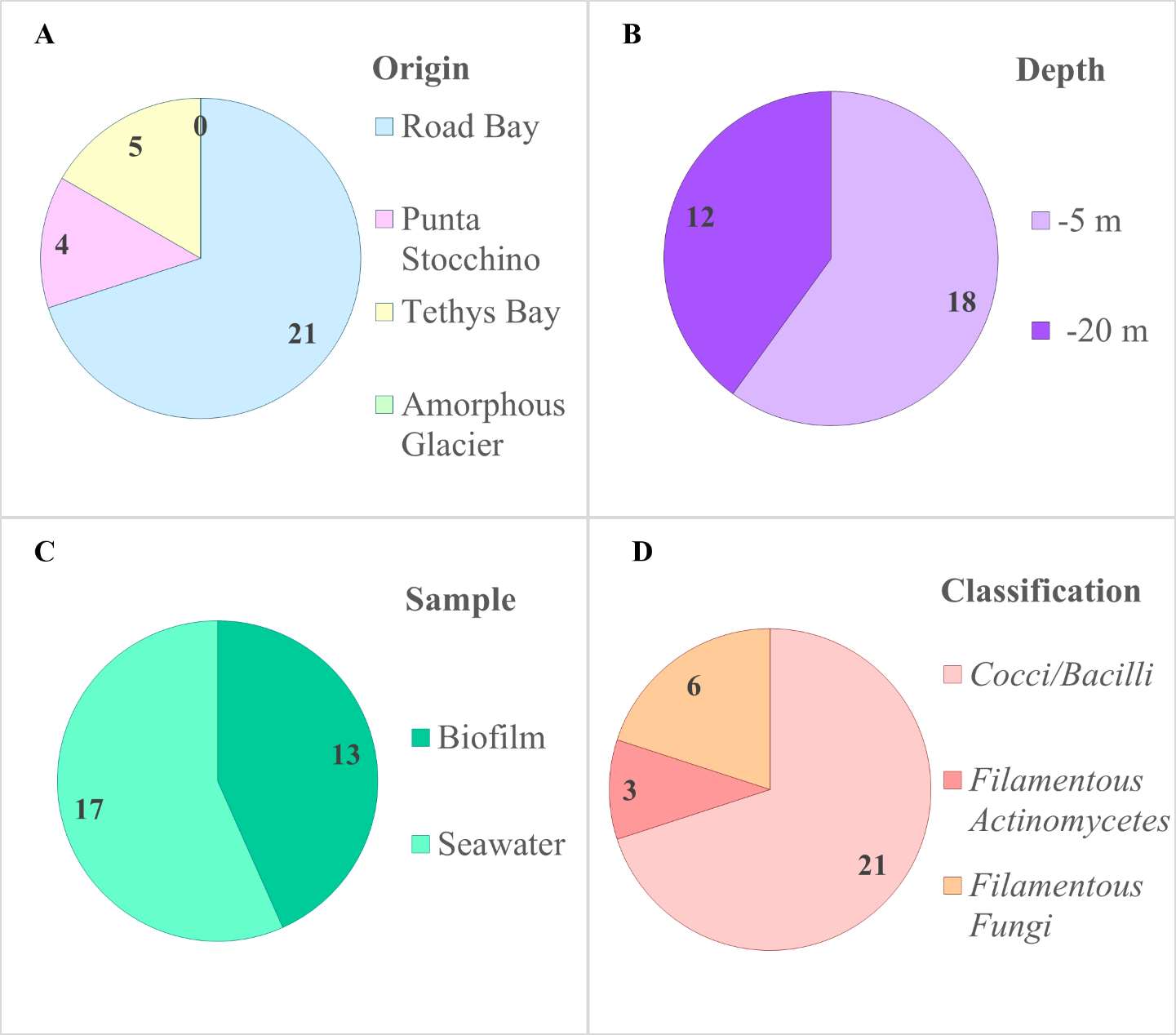

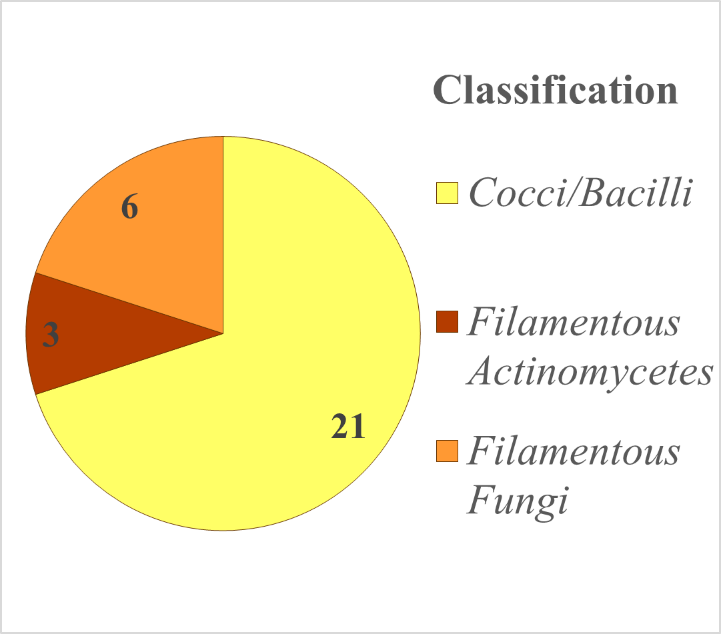


**D**

**Supplementary Figure 3.** Geographical distribution (A), sampling depth (B), sample type (C) and morphology-based classification (after dereplication, see Materials and Methods) (D) of the 30 microbial isolates from Antarctic biofilms and seawater samples (see Supplementary Figure 1 and 2), selected as positives in the primary screening and tested in the secondary screening for laccase activity.

**Supplementary Table B**

| **Strain** | **Environmental Sample** | **Isolation Media** | **Isolation**  **Temperature (°C)** | **Morphological Classification** | **Activity in solid medium on** | | **Activity in liquid on** | | | | | |
| --- | --- | --- | --- | --- | --- | --- | --- | --- | --- | --- | --- | --- |
|  |  |  |  |  | **ABTS** | **Azure B** | **Most Active Fraction** | **ABTS** | **2,6-DMP** | **Catechol** | **Azure B** |  |
| **B2** | Biofilm RB, PVC -5 m | Marine Agar | 28 | Cocci/Bacilli | + | - | C | + | - | - | - |  |
| **B10** | Biofilm PTS, PVC -5 m | Marine Agar | 28 | Cocci/Bacilli | + | - | C | + | - | - | - |  |
| **B15** | Biofilm RB, PVC -5 m | ISP5 | 28 | Filamentous  Fungi | + | + | S | + | - | + | + |  |
| **B20** | Biofilm RB, PVC -5 m | ISP4 | 20 | Cocci/Bacilli | - | + | C | - | - | - | + |  |
| **B25** | Biofilm RB, PVC -5 m | ISP4 +  Salt | 28 | Filamentous  Fungi | + | - | S | + | - | + | - |  |
| **B28** | Biofilm RB, PVC -5 m | ISP4 +  Salt | 20 | Filamentous  Actinomycete | - | + | S | - | - | - | + |  |
| **B29** | Biofilm RB, PVC -5 m | ISP4 +  Salt | 20 | Filamentous  Actinomycete | - | + | S | - | - | - | + |  |
| **B41** | Biofilm PTS, PVC -5 m | Chitin Agar | 4 | Filamentous  Fungi | + | + | S | + | - | - | + |  |
| **B43** | Biofilm RB, PVC -5 m | Chitin Agar | 4 | Filamentous  Fungi | + | + | S | + | - | - | + |  |
| **B46** | Biofilm PTS, PVC -5 m | ISP7 | 4 | Cocci/Bacilli | - | + | C | - | - | - | + |  |
| **B229** | Biofilm TB,  PE -5 m | Marine Agar | 20 | Filamentous  Fungi | - | + | S | - | - | - | + |  |
| **B233** | Biofilm PTS, PE -20 m | ISP4 + Salt | 20 | Filamentous  Fungi | + | + | S | + | - | - | + |  |
| **B255** | Biofilm RB, PVC -5 m | Marine Agar | 20 | Filamentous  Actinomycete | + | - | S | + | + | - | - |  |
| **M44** | Seawater  TB -20 m | Marine Agar | 5 | Cocci/Bacilli | + | - | C | + | - | - | - |  |
| **M45** | Seawater  TB -20 m | Marine Agar | 5 | Cocci/Bacilli | + | + | C | + | - | - | + |  |
| **M46** | Seawater  TB -20 m | Marine Agar | 5 | Cocci/Bacilli | + | + | C | + | - | - | - |  |
| **M47** | Seawater  TB -20 m | Marine Agar | 5 | Cocci/Bacilli | + | - | C | + | + | + | - |  |
| **M56** | Seawater  RB -5 m | Marine Agar | 5 | Cocci/Bacilli | + | + | C | + | - | + | - |  |
| **M60** | Seawater  RB -5 m | Marine Agar | 5 | Cocci/Bacilli | + | + | C | + | - | - | + |  |
| **M61** | Seawater  RB -5 m | Marine Agar | 5 | Cocci/Bacilli | + | - | C | + | + | - | - |  |
| **M62** | Seawater  RB -5 m | Marine Agar | 5 | Cocci/Bacilli | + | + | C | + | - | - | + |  |
| **M64** | Seawater  RB -5 m | Marine Agar | 5 | Cocci/Bacilli | + | + | C | + | - | - | + |  |
| **M65** | Seawater  RB -5 m | Marine Agar | 5 | Cocci/Bacilli | + | + | C | + | - | - | + |  |
| **M66** | Seawater  RB -20 m | Marine Agar | 5 | Cocci/Bacilli | + | + | C | + | - | - | + |  |
| **M67** | Seawater  RB -20 m | Marine Agar | 5 | Cocci/Bacilli | + | - | C | + | - | - | - |  |
| **M68** | Seawater  RB -20 m | Marine Agar | 5 | Cocci/Bacilli | + | + | C | + | + | - | - |  |
| **M70** | Seawater  RB -20 m | Marine Agar | 5 | Cocci/Bacilli | + | + | C | + | + | - | + |  |
| **M71** | Seawater  RB -20 m | Marine Agar | 5 | Cocci/Bacilli | + | - | C | + | - | - | - |  |
| **M73** | Seawater  RB -20 m | Marine Agar | 5 | Cocci/Bacilli | + | + | C | + | + | - | + |  |
| **M74** | Seawater  RB -20 m | Marine Agar | 5 | Cocci/Bacilli | + | - | C | + | - | - | - |  |

**Supplementary Table B.** Antarctic strains which were found active in the primary screening (solid agar plates) and were tested in the secondary screening (liquid cultures) for laccase activities. Primary screening was conducted on MAM added with 2% w/v artificial sea salt and supplemented with 5 mM ABTS or 25 mg/L azure B. Secondary screening was conducted on both supernatants (S) and crude extracts after cell lysis (C) (in the table the most active sample between the two is indicated) from microbial isolates grown in liquid media (these last selected according to the morphological classification of the isolates, see Material and Methods) using a wider range of substrates (1 mM ABTS, 1 mM 2,6-DMP, 10 mM catechol, 0.05 mM azure B, 0.05 mM Remazol brilliant blue R, 0.05 mM reactive black 5). + indicates presence of activity, - absence of activity. For each microorganism, information about the environmental sample from which it was isolated (marine biofilm-B or seawater sample-M), the site where the sample was collected (Road Bay-RB, Punta Stocchino-PTS, Tethys Bay-TB, Amorphous Glacier-AG) and its depth (-5 or -20 m) is indicated. Isolation media and isolation temperatures, and morphological-based classification are also reported.

**Supplementary Table C**

| **Selected strains** | **Colony Morphology** | **Gram Staining** | **Microscopic Morphology** | **Oxidase Activity** | **Glucose Fermentation** | **Leucine Aminopeptidase-** | **α-/β-Glucosidase-** | **Alkaline Phosphatase-** |
| --- | --- | --- | --- | --- | --- | --- | --- | --- |
|  |  |  |  |  |  | **Activity** | | |
| **M47** | Compact with yellow pigmentation | Negative | Cocci | + | - | + | - | + |
| **M61** | Compact | Positive | Cocci | + | -  (oxidizing) | + | + (α) | + |
| **M68** | Compact with orange pigmentation | Negative | Bacilli | +/- | -  (oxidizing) | + | - | + |
| **M70** | Compact | Negative | Vibrio | + | -  (oxidizing) | + | - | + |
| **M73** | Compact | Positive | Cocci | + | - | + | - | + |

**Supplementary Table C.** Phenotypical and enzymatic characterization of the five selected unicellular bacteria from seawater samples. Colony morphology was observed after 15-20 days growth in Marine Agar. Gram staining was determined using the Oxoid Gram staining kit following described procedures (Caruso et al, 2022). Morphology was observed at optical microscopy (Zeiss Primo Star, 40x magnification). Oxidase activity was determined by converting the Kovacs colorless reagent in a colored product (Shields and Cathcart, 2010). Glucose fermentation was tested by Triple Sugar Iron (TSI) test (Oxoid): oxidizing refers to strains performing the oxidative decarboxylation of peptones (alkaline reaction causing the medium colour to turn dark red) but no glucose fermentation (Hajna, 1945). The enzymatic activities were detected using fluorescent substrates specific for leucine aminopeptidase, α- and β-glucosidases, alkaline phosphatases as reported in (Kim and Hoppe, 1986).


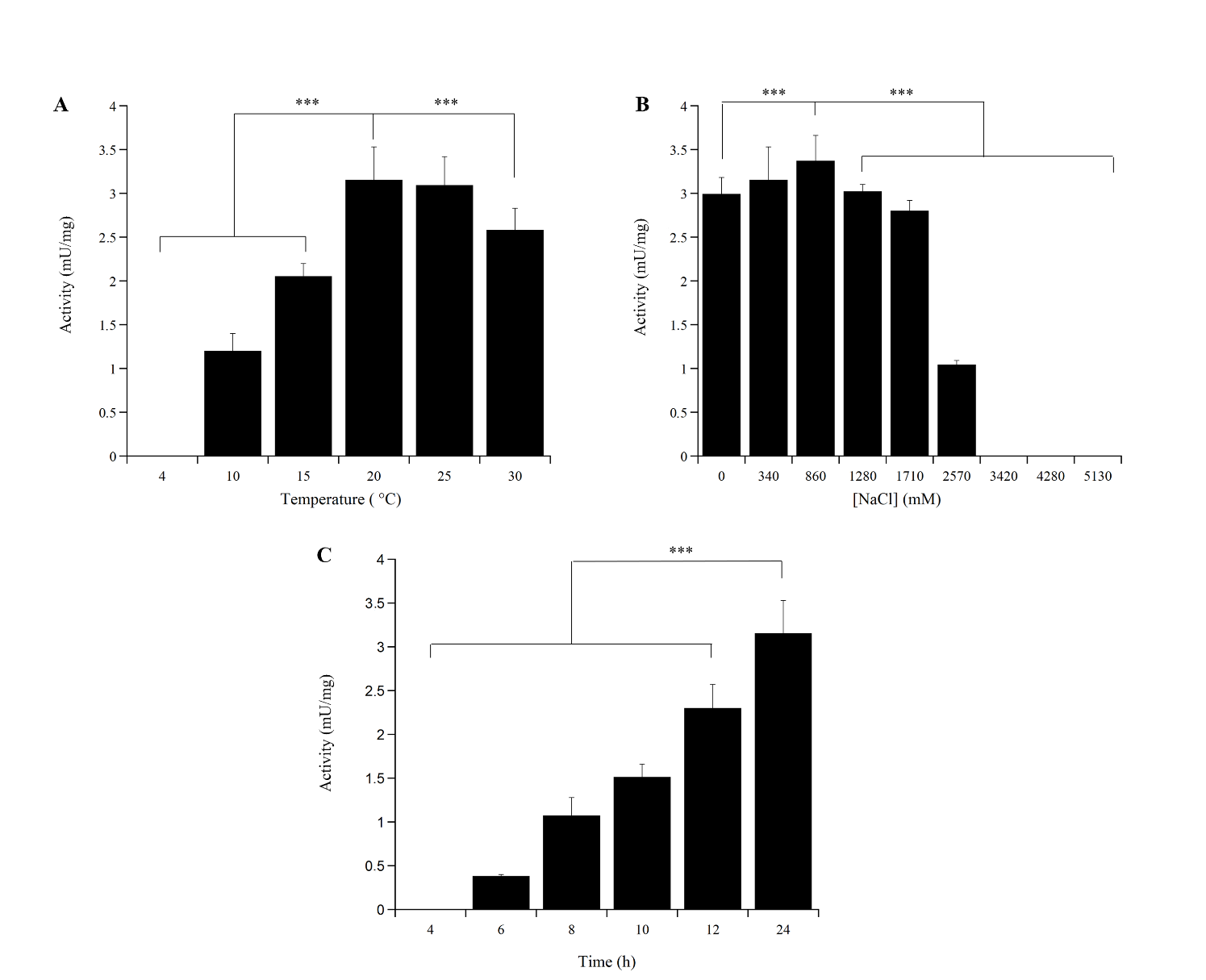


**Supplementary Figure 4.** Effect of the growth temperature (A), NaCl concentration (B) and cultivation time (C) on Ant laccase production from *Halomonas* sp. M68. Enzyme activity was measured on 5 mM ABTS in 50 mM sodium acetate pH 5.0. Values represent the means of three independent experiments (mean ± standard deviation). The results were evaluated by statistical analysis using one-way ANOVA followed by a Tukey’s multiple comparison test. ****p*<0.0001.


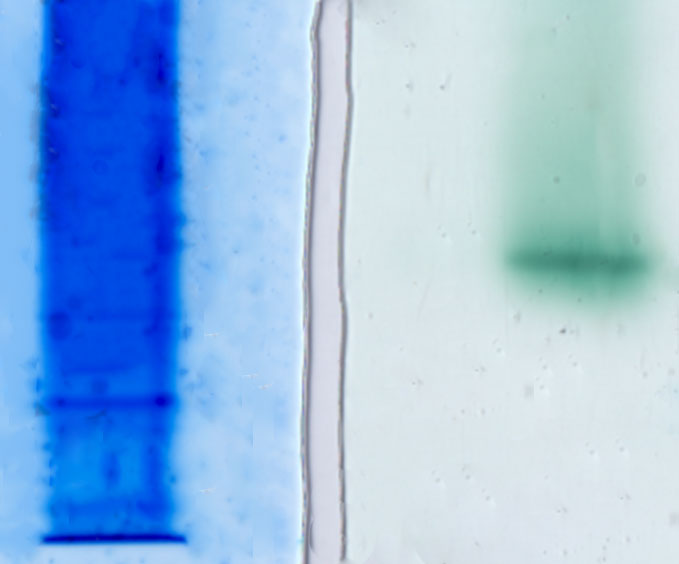


**(B)**

**(A)**

**Supplementary Figure 5.** Electrophoretic analysis of crude enzyme preparation from *Halomonas* sp. M68 (SNP30). Native-PAGE analysis of the sample with two different staining procedures: (A) Coomassie-like staining and (B) laccase activity staining (on 10 mM ABTS in 50 mM sodium acetate, pH 5.0 at room temperature).


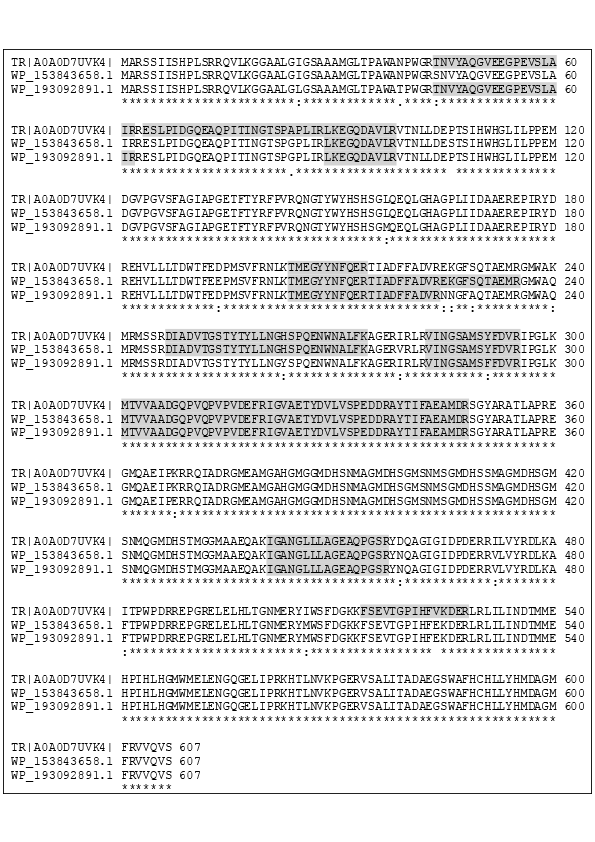


**Supplementary Figure 6.** Multiple sequence alignment of copper resistance protein CopA and copper resistance system multicopper oxidases using ClustalW. Peptide sequences identified by nLC-MS/MS analysis are highlighted in grey. * (asterisk) indicates positions which have a single, fully conserved residue and: (colon) indicates conservation between groups of residues with strongly similar properties.


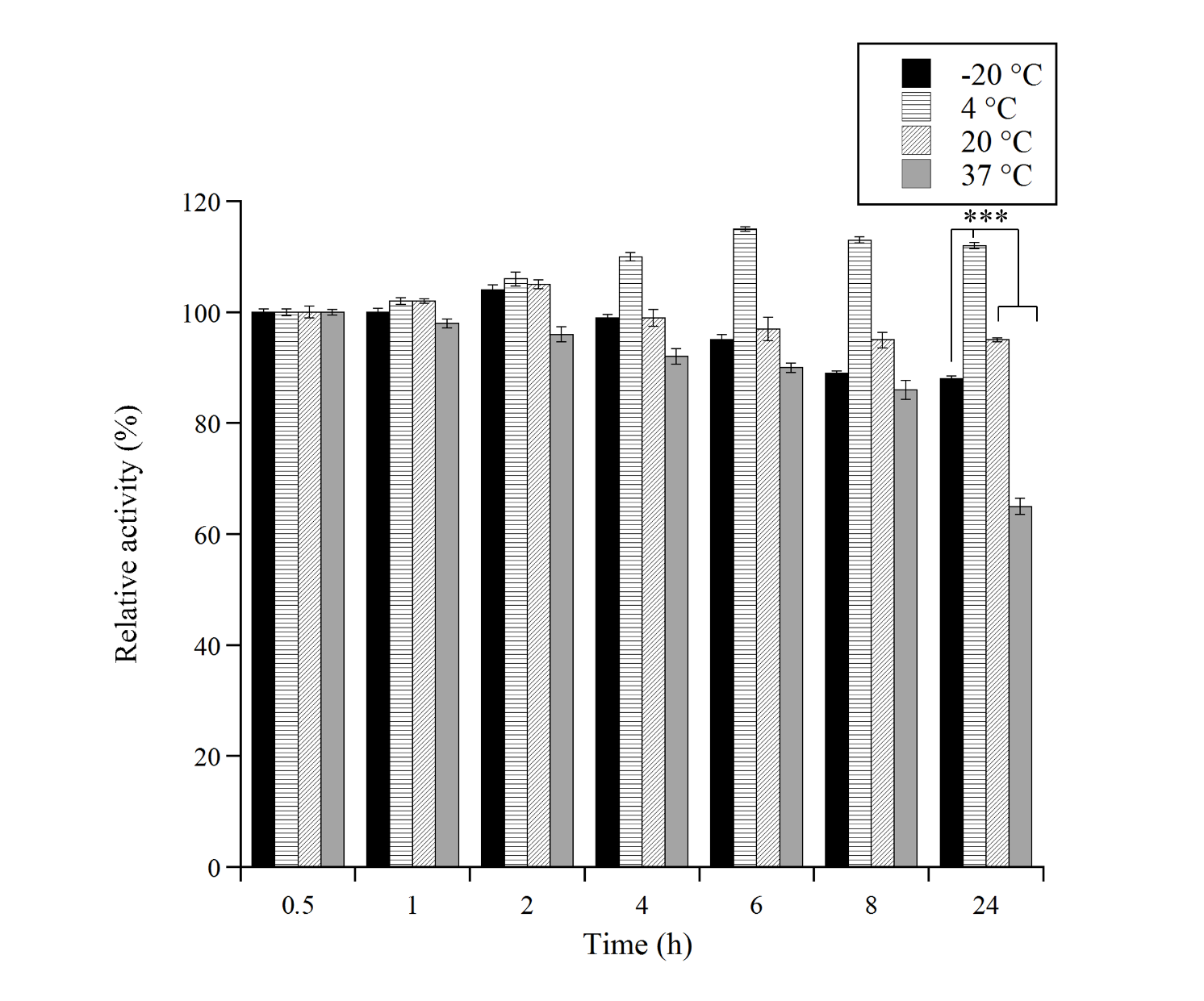


**Supplementary Figure 7.** Effect of the incubation temperature on the stability of the Ant laccase preparation from *Halomonas* sp. M68 (SNP30) determined by measuring ABTS oxidation. The residual activity was measured on 5 mM ABTS in 50 mM sodium acetate, pH 5.0 within 24 h of incubation at different temperatures. The activity value at time=0 for each temperature was taken as 100%. Values represent the means of three independent experiments (mean ± standard deviation). The results were evaluated by statistical analysis using one-way ANOVA followed by a Tukey’s multiple comparison test. ****p*<0.0001.

**References**

Baxter M., Sieburth J. M. (1984). Metabolic and ultrastructural response to glucose of two eurytrophic bacteria isolated from seawater at different enriching concentrations. *Appl. Environ. Microbiol..* 47(1), 31-38. doi:10.1128/aem.47.1.31-38.1984

Caruso, G., Dell’Acqua, O., Caruso, R., Azzaro, M. (2022). Phenotypic characterization of bacterial isolates from marine waters and plastisphere communities of the Ross Sea (Antarctica). *J. Clin. Microbiol. Biochem. Technol.* 8(1), 1-9. doi: 10.17352/jcmbt.000048

Hajna, A. A. (1945). Triple-sugar iron agar medium for the identification of the intestinal group of nacteria. *J. Bacteriol.* 49(5), 516-517. doi: 10.1128/jb.49.5.516-517.1945

Hsu, S. C., Lockwood, J. L. (1975). Powdered chitin agar as a selective medium for enumeration of actinomycetes in water and soil. *Appl. Microbiol.* 29(3), 422-426. doi: 10.1128/am.29.3.422-426.1975

Kim, S. J., Hoppe, H. G. (1986) Microbial extracellular enzyme detection on agar plates by means of ﬂuorogenic methylumbelliferyl-substrates. *GERBAM-CNRS Actes des Colloques* 3, 175–183.

Marcone, G. L., Binda, E., Reguzzoni, M., Gastaldo, L., Dalmastri, C., Marinelli, F. (2017). Classification of *Actinoplanes* sp. ATCC 33076, an actinomycete that produces the glycolipodepsipeptide antibiotic ramoplanin, as *Actinoplanes ramoplaninifer* sp. nov. *Int. J. Syst. Evol. Microbiol.* 67(10), 4181-4188. doi:10.1099/ijsem.0.002281

Reddy, G. S., Aggarwal, R. K., Matsumoto, G. I., Shivaji, S. (200) *Arthrobacter flavus* sp. nov., a psychrophilic bacterium isolated from a pond in McMurdo Dry Valley, Antarctica. *Int. J. Syst. Evol. Microbiol.* 50(4), 1553-1561. doi:10.1099/00207713-50-4-1553

Shields, P., Cathcart, L. (2010). Oxidase test protocol. *American Society for Microbiology.*
